# Programmable chalcogenide-based all-optical deep neural networks

**DOI:** 10.1515/nanoph-2022-0099

**Published:** 2022-05-25

**Authors:** Ting Yu Teo, Xiaoxuan Ma, Ernest Pastor, Hao Wang, Jonathan K. George, Joel K. W. Yang, Simon Wall, Mario Miscuglio, Robert E. Simpson, Volker J. Sorger

**Affiliations:** Singapore University of Technology and Design, 8 Somapah Road, Singapore 487372, Singapore; Deptartment of Electrical and Computer Engineering, George Washington University, Washington, DC, USA; ICFO - Institut de Ciencies Fotoniques, The Barcelona Institute of Science and Technology, Av. Carl Friedrich Gauss 3, Castelldefels 08860, Barcelona, Spain; Department of Physics and Astronomy, Aarhus University, Ny Munkegade 120, Aarhus C 8000, Denmark

**Keywords:** all-optical deep neural network, chalcogenide reconfigurable photonics, ultra-fast dynamic response of phase change material

## Abstract

We demonstrate a passive all-chalcogenide all-optical perceptron scheme. The network’s nonlinear activation function (NLAF) relies on the nonlinear response of Ge_2_Sb_2_Te_5_ to femtosecond laser pulses. We measured the sub-picosecond time-resolved optical constants of Ge_2_Sb_2_Te_5_ at a wavelength of 1500 nm and used them to design a high-speed Ge_2_Sb_2_Te_5_-tuned microring resonator all-optical NLAF. The NLAF had a sigmoidal response when subjected to different laser fluence excitation and had a dynamic range of −9.7 dB. The perceptron’s waveguide material was AlN because it allowed efficient heat dissipation during laser switching. A two-temperature analysis revealed that the operating speed of the NLAF is ≤1 ns. The percepton’s nonvolatile weights were set using low-loss Sb_2_S_3_-tuned Mach Zehnder interferometers (MZIs). A three-layer deep neural network model was used to test the feasibility of the network scheme and a maximum training accuracy of 94.5% was obtained. We conclude that combining Sb_2_S_3_-programmed MZI weights with the nonlinear response of Ge_2_Sb_2_Te_5_ to femtosecond pulses is sufficient to perform energy-efficient all-optical neural classifications at rates greater than 1 GHz.

## 1 Introduction

With the advent of artificial intelligence, computers are tasked to mimic the biological brain and perform data-centric tasks, like image and speech recognition. However, implementing and training artificial neural networks (ANN) are computationally intensive when large amounts of data are being processed. Current computing technologies are not optimized to efficiently perform data-centric tasks. It is estimated that by 2040, computers will require more energy than we could generate to process the data created [1]. Hence, faster and more energy-efficient computing hardware must be developed to cope with this demand.

New computing paradigms have emerged in recent years to enhance computer performance. One promising solution transforms the virtual ANN machine learning algorithm into hardware. Neural-inspired application specific hardware devices can accelerate multiply-and-accumulate (MAC) operations, which constitutes the weighting functionality of an ANN. On-chip neural-inspired weights have been extensively developed [2], [3], [4]. These devices are usually implemented in the optical domain to avoid electrical circuit inefficiencies like lossy electrical interconnects and capacitive charging [5].

Despite the recent advances in neural-inspired optical weighting devices, the other necessary ANN components are still reliant on current computing technologies. This includes the nonlinear activation function (NLAF) [2], [3], [4], which is essential for network training and the decision mapping process. Data shuttling between the hardware weights and virtual ANN components will compromise network speed and energy usage. There have been efforts to develop the NLAF device. However, current hardware NLAF components require further optimization as they have not been demonstrated on a device level [6], require electrical to optical conversion [7], are inefficient in terms of speed or/and energy usage [8, 9], or have complicated fabrication processes [10].

To develop a full hardware neural network, the next design iteration involves efficiently integrating the NLAF with the weights. Integrating both components together is critical as they constitute the perceptron, which is the basic building block of a neural network. Devices within the perceptron must exhibit both nonvolatile and volatile tunability. The weighting components must be nonvolatile to retain programmed weights when performing MAC operations. Meanwhile, the NLAF device must be volatile as it needs to retain its original mapping function when processing each incoming signal.

Using chalcogenide phase change material (PCM)-based reconfigurable photonic devices to develop an all-optical perceptron is desirable as conventional photonic waveguide devices can now become tunable and perform switching operations. Although structural phase transitions in chalcogenides have already been proposed to tune the optical neural networks weighted interconnections [3, 11], they have not been studied for performing nonlinear activation. Chalcogenides are known to exhibit a range of nonlinear optical effects, especially third order nonlinearity, $\chi^{3}$ [12]. These nonlinearities could be used to implement the NLAF in an all-optical perceptron.

Besides the third order nonlinearity, $\chi^{3}$ , the highly nonlinear volatile change to the optical constants of crystalline Ge_2_Sb_2_Te_5_ , which was previously reported, could be important for creating NLAFs [13, 14]. The *p*-orbital electrons in some crystalline chalcogenides, such as Ge_2_Sb_2_Te_5_, can be delocalized and therefore highly polarizable, which enhances the material’s dielectric permittivity. This was demonstrated by Shportko et al. where there was a large increase in electronic polarizability when the PCM changed from the amorphous to crystalline state [15]. Several studies suggest that this permittivity enhancement can be temporarily disrupted by an external stimulus, such as short laser or electrical pulses [13, 16], [17], [18]. We hypothesized that this high-speed volatile change in dielectric permittivity could be exploited to generate a NLAF. Moreover, by combining structural transitions to set the network’s nonvolatile weights with the Ge_2_Sb_2_Te_5_ nonlinear activation function, we further hypothesized that feed-forward neural network inference operations would be possible without converting the optical signal into the electrical domain.

Herein, we designed, modeled, and optimized a low-loss all-optical chalcogenide perceptron that can recognize images using a convolutional neural network approach. In the proposed all-chalcogenide optical perceptron model, both the weights and the NLAF consist of chalcogenide reconfigurable devices. The perceptron device consists of a low-loss Sb_2_S_3_-tuned MZI, which is used to set the network weights, and a sigmoidal NLAF using a crystalline Ge_2_Sb_2_Te_5_-tuned ring resonator. We experimentally show volatile switching of crystalline Ge_2_Sb_2_Te_5_, with the optical properties being tuned within 1 ps when excited with a femtosecond laser pulse. For the first time, this transient optical response of Ge_2_Sb_2_Te_5_ was modeled on a photonic device to implement a sigmoidal NLAF. Based on our simulation model, the sigmoidal NLAF has a dynamic range of 9.7 dB. Using a two-temperature model to describe how femtosecond excitation can influence the NLAF transmission, we show that the perceptron can have an overall computation speed of more than 1 GHz in a feedforward neural network scenario. An optical neural network was modeled using the proposed all-chalcogenide-tuned perceptron design. Network performance accuracy of up to 94.5% was achieved when used to infer characters from the MNIST dataset.

## 2 Materials and methods

### 2.1 Ge_2_Sb_2_Te_5_ material fabrication

Amorphous Ge_2_Sb_2_Te_5_ thin films were deposited on glass substrates by radio frequency magnetron sputtering using an AJA Orion 5 sputtering system with a base pressure of 2.5 × 10^−7^ Torr. The Ge_2_Sb_2_Te_5_ material comes from a commercially purchased target with diameter of 50.8 mm and purity of 99.9%. The sputtering process took place in an argon environment at a pressure of 3.7 × 10^−3^ Torr. The RF power was set to 30 W, which resulted in a deposition rate of 0.093 Å/s. The deposition rate was calibrated using step profilometry of films deposited for a fixed time.

To crystallize the amorphous Ge_2_Sb_2_Te_5_ thin film, we first determined the phase transition temperature. This was done by recording the change in optical reflectivity as we heated the material. The first abrupt change in reflectivity of the thin film indicates an amorphous to face-centered cubic (FCC) structural phase transition [19]. The as-deposited amorphous Ge_2_Sb_2_Te_5_ thin film was heated to 250 °C, at a heating rate of 5 °C/min in a Linkam heating stage (Linkam T95-HT). To prevent oxidation, Ar gas at 4 SCCM was supplied to the heating enclosure. The material reflectivity was recorded each time the heating stage temperature increased by 1 °C. To ensure consistency, this sample was from the same sputtering batch as the sample to be used in the femtosecond laser switching experiment. The phase transition temperature was found to be at 176 °C. Upon determining the phase transition temperature, we then crystallize the actual amorphous Ge_2_Sb_2_Te_5_ sample used in the femtosecond laser switching experiment. To ensure that the material was fully crystallized, we heated the sample to 183 °C with a heating ramp rate of 5 °C/s and a hold time of 30 min. Similarly, an Ar gas flow of 4 SCCM was supplied to the heating enclosure to prevent oxidation. The reflectivity measurements of the Ge_2_Sb_2_Te_5_ film can be found in the Supplementary Material Section 1.

### 2.2 Time-resolved femtosecond switching experiment

The pump–probe transient transmission measurements were performed on the crystalline Ge_2_Sb_2_Te_5_ at a wavelength of 1500 nm after pumping with an 800 nm pulse of 35 fs duration. A commercial regenerative 5 kHz Ti:Sapphire amplifier (Coherent) generated the 800 nm pump pulses, which were split and sent to the sample and to a commercial OPA (Light conversion TOPAS) to generate the probe pulse (1500 nm). To ensure a uniform excitation area, the pump spot (FWHM 180 × 100 µm) was significantly larger than the probe. A mechanical chopper at 500 Hz was used to modulate the pump and the changes in reflectance, $\frac{\Delta R_{\exp}}{R_{\exp}}$ , and in transmittance, $\frac{\Delta T_{\exp}}{T_{\exp}}$ , with respect to time were simultaneously recorded using two InGaAs detectors. Note, subscript “exp” represents the experimental measurements. We derived the corresponding changes in the optical constants (refractive index *n* and dielectric function *k*) from the measured $\frac{\Delta R_{\exp}}{R_{\exp}}$ and $\frac{\Delta T_{\exp}}{T_{\exp}}$ using a procedure previously reported [13]. In brief, the procedure involved calculating the changes in transmission:

(1) $$\begin{array}{c} \frac{\Delta T_{\mathrm{calc}}}{T_{\mathrm{calc}}}\left(n^{\prime },d,\lambda \right)=\frac{T\left(n^{\prime },d,\lambda \right)}{T\left(n_{0},d,\lambda \right)}-1 \end{array}$$

and reflection:

(2) $$\begin{array}{c} \frac{\Delta R_{\mathrm{calc}}}{R_{\mathrm{calc}}}\left(n^{\prime },d,\lambda \right)=\frac{R\left(n^{\prime },d,\lambda \right)}{R\left(n_{0},d,\lambda \right)}-1 \end{array}$$

for the multilayer structure (Ge_2_Sb_2_Te_5_/SiO_2_ in our case) using a transfer matrix method described by Born et al. [20] at a given wavelength, *λ*, thickness, *d*. Moreover, *n*_0_ = 6.5 + 1.2*i* and *n*′ are the initial and perturbed complex refractive indices, respectively. Subsequently, we fitted the change in transmittance and reflectance, $\frac{\Delta T_{\mathrm{calc}}}{T_{\mathrm{calc}}}$ and $\frac{\Delta R_{\mathrm{calc}}}{R_{\mathrm{calc}}}$ , to the measured experimental data, $\frac{\Delta T_{\exp}}{T_{\exp}}$ and $\frac{\Delta R_{\exp}}{R_{\exp}}$ , and obtained the values of $n^{\prime }$ that gave the best fit to both datasets simultaneously. This allowed us to obtain a unique solution for the optical constant values of Ge_2_Sb_2_Te_5_. The analysis was performed at each probe delay. Multiple reflections in the SiO_2_ substrate were ignored. The effects of small variations in the Ge_2_Sb_2_Te_5_ thicknesses (24, 27 and 30 nm, respectively) were found to only give a small error in the retrieved refractive index. To account for the change in material thickness upon crystallization [21], our analysis and simulation models used the mean value of the optical constants derived from the three thickness values.

### 2.3 Waveguide design and modeling

#### 2.3.1 Ring resonator

The Microring Resonator (MRR) NLAF spectrum was calculated using the power transfer function [22]:

(3) $$\begin{array}{c} \left|S_{21}\right|=\frac{\upsilon^{2}-2\upsilon \xi \;\text{cos}\;\phi +\xi^{2}}{1-2\upsilon \xi \;\text{cos}\;\phi +{\left(\upsilon \xi \right)}^{2}} \end{array}$$

where υ is the self-coupling coefficient, ϕ is the phase shift and ξ is the amplitude attenuation coefficient for one round trip in the ring.

For the Ge_2_Sb_2_Te_5_-tuned ring resonator, ϕ and ξ can be expressed as [23]:

(4) $$\begin{array}{c} \phi =\frac{2\pi }{\lambda }n_{\text{eff},\text{AlN}}\left(L-x\right)+\frac{2\pi }{\lambda }n_{\text{eff},\text{GST}}\left(x\right) \end{array}$$

(5) $$\begin{array}{c} \xi ={\text{e}}^{-\frac{\alpha_{\text{AlN}}\left(L-x\right)+\alpha_{\text{GST}}\left(x\right)}{2}} \end{array}$$

where *λ* is the wavelength, *n*_eff_ is the effective refractive index, α is the absorption coefficient, *L* is the ring length and *x* is the length of the Ge_2_Sb_2_Te_5_.

The *n*_eff_ values and $\alpha_{\text{GST}}$ were calculated using the eigenmode solver in Lumerical Mode Solution. The corresponding *n*_eff_ values and mode profile of the AlN waveguide and Ge_2_Sb_2_Te_5_-based AlN waveguide can be found in the Supplementary Material Section 2. Optical constants used in the simulation model were as follows: AlN waveguide and SiO_2_ substrate were from references [24, 25] and Ge_2_Sb_2_Te_5_ was from Figure 3D and E. The $\alpha_{\text{AlN}}$ value was set to be 0.8 dB/cm as reported in reference [26].

υ is expressed as:

(6) $$\begin{array}{c} \upsilon^{2}+m^{2}=1-\text{loss} \end{array}$$

where *m* is the cross-coupling coefficient and the loss represents the losses in the coupling section.

The AlN ring resonator spectrum reported in reference [26] was replicated using Eqs. (3)–(5) to estimate the loss value in the coupling section expressed in Eq. (6). *m* was derived by modeling the coupling region on Lumerical and performing 3D Finite Difference Time Domain (3D FDTD) calculations.

#### 2.3.2 Directional coupler

The design of the Sb_2_S_3_ directional coupler for the MZI weights was reported in references [27, 28]. The optical constants used for the waveguide and substrate were identical to the MRR model and the Sb_2_S_3_ optical constants were from reference [28]. The optimized dimensions of the directional coupler and the mode profile of the AlN waveguide and Sb_2_S_3_-based AlN waveguide can be found in the Supplementary Material Section 3.

## 3 Results and discussion

### 3.1 All-optical perceptron design

**Figure 1:**
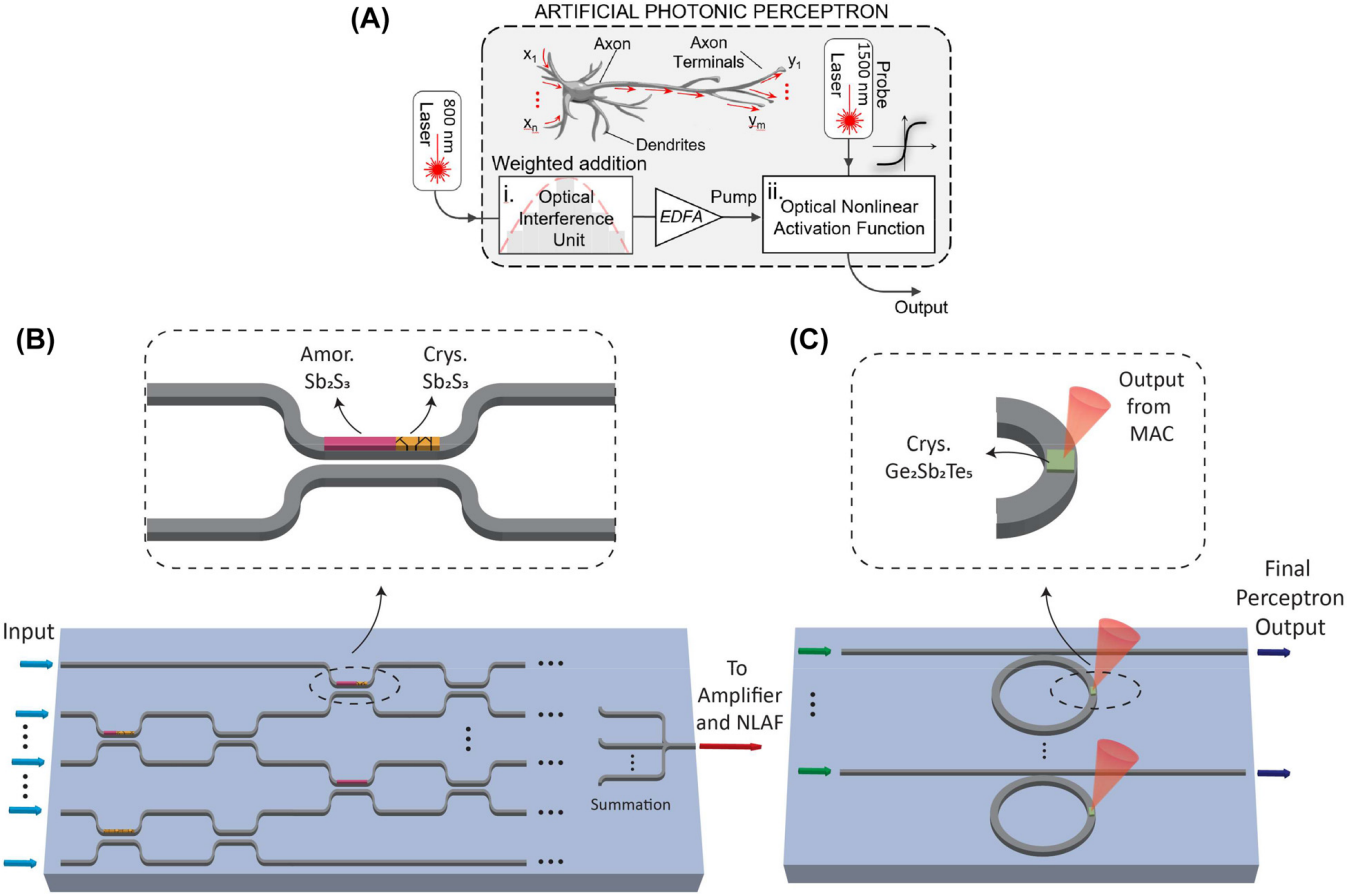
All-chalcogenide optical perceptron model. (A) Schematic diagram of the perceptron. (B) P^3^A consisting of a network of reprogrammable MZI weights. Reconfigurability is introduced by the Sb_2_S_3_ - tuned directional coupler arm. (C) Ge_2_Sb_2_Te_5_-tuned MRR NLAF.

The proposed all-chalcogenide perceptron is shown in Figure 1A. The multiplication operation is performed by a photonic programmable phase-change array (P^3^A) as shown in Figure 1B, which consist of a network of reprogrammable MZI. In the MZI, one of the coupling waveguides in the input directional coupler arm contains a strip of PCM; as shown in the inset of Figure 1B. The amorphous to crystal ratio of the PCM strip is tuned to represent different weight values [28]. The resultant output signal from each MZI is then summed together when the waveguides converge to one point. The optical output of the MAC operation is then amplified to program the PCM on the NLAF component. Amplification is done in the optical domain using either an on-chip semiconductor optical amplifiers (SOAs) or Erbium Doped Fiber Amplifiers (EDFAs). Figure 1C shows the NLAF array made from MRRs. The final output of the perceptron is an optical signal that propagates through the NLAF component as the PCM NLAF is simultaneously programmed by the weights. Note that the optical input of the NLAF, as indicated by the green arrow in Figure 1C, is a separate probe signal from the MAC output.

Optimizing the PCM for each component is critical for efficient network performance. The PCM in the weighting component must retain its optical property. It must also be low loss to allow network scalability. Common PCM data storage materials, such as Ge_2_Sb_2_Te_5_, are too absorbing in both the visible and infrared. Recently, a new generation of wide bandgap PCMs were introduced [29, 30] and their optical losses are lower than the Te-based PCM compounds. Sb_2_S_3_ was reported to have the largest bandgap in the visible and N-IR amongst the emerging PCMs [28, 31], which corresponds to the lowest optical absorption. We recently showed that Sb_2_S_3_ is the most promising candidate material for programming optical directional couplers [28] due to its low-loss property. Moreover, Sb_2_S_3_ can be programmed with different degrees of crystallinity by partial amorphization [32]. For these reasons, we chose Sb_2_S_3_ as the PCM to tune the weights of the P^3^A.

The PCM in the NLAF component must exhibit a nonlinear change in optical properties and volatile tunability. Whilst chalcogenides tend to exhibit a range of nonlinear optical effects, for instance $\chi^{3}$ , it may be challenging to implement them on nanophotonic platforms. This is because long optical path lengths are required for phase accumulation. In contrast, Tellurides, in just tens of nm thickness, tend to show nonlinear changes in optical properties when the delocalized *p*-orbital bonds in the crystalline structure are disrupted. The delocalized *p*-orbital bonds enhance the optical matrix elements. This was attributed to resonant bonding [15] but more recently distinct difference to textbook resonant bonding have been highlighted, and metavalent bonding [33] and, hyperbonding [34] have been used to describe the root cause of these unusually large optical matrix elements. Importantly, delocalized *p*-orbital bonds in crystalline Ge_2_Sb_2_Te_5_ are strongly disturbed by femtosecond laser pulses [13]. The femtosecond laser pulse changes the optical properties of crystalline GeTe–Sb_2_Te_3_ alloys without perturbing the lattice until much later, in the picosecond time range. Moreover, the material can return to its original crystalline optical state when it does not accumulate sufficient heat energy from the laser pulse to amorphize it. This suggests that Sb_2_Te_3_–GeTe materials can perform nonlinear optical volatile tunability. The delay in lattice perturbation is also an advantage as it reduces switching variability. This is because the refractive index of the waveguide will vary when subjected to thermal expansion. By delaying lattice perturbation, the optical signal will not be affected by a change in refractive index of the waveguide during the femtosecond laser switching process. Moreover, since Ge_2_Sb_2_Te_5_ does not change structural phase, stress is minimized, and we expect the cyclability endurance to be higher than that of phase change-tuned materials. Therefore, we propose to use femtosecond laser switching of Ge_2_Sb_2_Te_5_ to implement a purely optical NLAF.

Integrating both PCM-based neural network components on the same chip requires the photonic platform to support both functionalities. Thus, optimizing the waveguide material of the chip becomes critical. The waveguide material should exhibit a large bandgap to support visible and near infrared (N-IR) operations. This is because the weighting component must operate in the visible region as the Ge_2_Sb_2_Te_5_ NLAF component requires an 800 nm wavelength femtosecond optical signal for switching [13]. Moreover, the NLAF component is set to operate in the telecommunication wavelength (N-IR region) as it can be easily integrated with other Si or SiN-based photonic components commonly found on a photonic integrated chip (PIC). In addition, operating in the N-IR wavelength is more efficient as Ge_2_Sb_2_Te_5_ exhibits lower optical losses in the near infrared compared to the visible [28]. The waveguide material should also be optimized to efficiently dissipate heat from the Ge_2_Sb_2_Te_5_ material to minimize network latency. Hence, waveguide materials that have a high heat conductivity are necessary.

**Figure 2:**
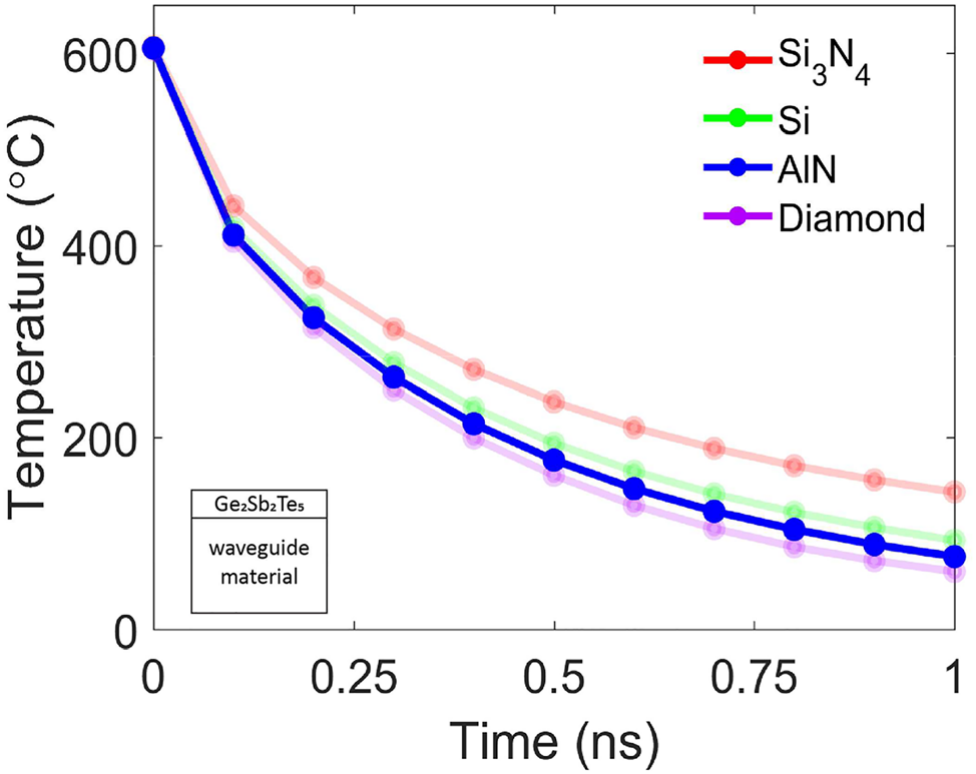
Temperature variation of the Ge_2_Sb_2_Te_5_ film with time as it cools. Figure inset shows the Ge_2_Sb_2_Te_5_/waveguide stack simulated. More information of the simulation parameters can be found in the Supplementary Material Section 4.

To choose an appropriate waveguide material for this setup, we compared the properties of four prototypical waveguide materials: Si, SiN, AlN and diamond [35–38]. Table 1 shows the optical and thermal properties of the waveguide materials. A Ge_2_Sb_2_Te_5_/waveguide stack shown in the inset of Figure 2 was modeled to understand how the waveguide material affects Ge_2_Sb_2_Te_5_ heat dissipation. In the model, we set the Ge_2_Sb_2_Te_5_ layer to be at 615 °C, which is its melting point, and let the structure cool to room temperature. The temporal variation in temperature of the Ge_2_Sb_2_Te_5_ layer was modeled using the finite element methods (FEM) on COMSOL to solve the heat diffusion equation. More information on the simulation parameters can be found in the Supplementary Material Section 4. From the simulation, we found that the waveguide material could no longer improve the heat dissipation rate of the Ge_2_Sb_2_Te_5_ layer when its thermal conductivity is in the range of 10^2^–10^3^ *W*/*m*⋅*K*. Instead, the heat dissipation rate is mostly limited by the Ge_2_Sb_2_Te_5_ layer itself. This is evident in Figure 2, where the Ge_2_Sb_2_Te_5_/diamond stack was only 32 °C lower than the Ge_2_Sb_2_Te_5_/Si stack at 1 ns whilst the Ge_2_Sb_2_Te_5_/Si stack was 50 °C lower than the Ge_2_Sb_2_Te_5_/SiN stack. Note, the thermal conductivity of diamond is an order of magnitude higher than Si whilst thermal conductivity of Si is only 5 times higher than SiN. Hence the waveguide materials Si, AlN and diamond are suitable to efficiently dissipate heat from the Ge_2_Sb_2_Te_5_ layer. However, Si does not transmit visible light and diamond is not compatible with CMOS processing. Moreover, complex fabrication processes are required to obtain single-crystal diamond waveguides that exhibit the superior thermal properties found in Table 1. Ultimately, we chose AlN as the waveguide material due to its large bandgap, ability to transmit in the visible and N-IR, low thermo-optic coefficient, CMOS compatibility, and its ability to efficiently dissipate heat from the Ge_2_Sb_2_Te_5_ material.

**Table 1:** Comparison of optical and thermal properties of waveguide materials.

Waveguide material	Refractive index at 1550 nm	Transparency wavelength region (µm)	CMOS compatibility	Thermal conductivity (*W*/*m*⋅*K*)	Thermo-optic coefficient (/K)
Si [39–41]	3.47	1.2–8	Yes	131	1.8 × 10^−4^ (@ 1550 nm)
SiN [39, 42, 43]	2.0	0.4–4.6	Yes	20	2.45 × 10^−5^ (@ 1550 nm)
AlN [35, 39, 44, 45]	2.12	0.2–13.6	Yes	285	2.3 × 10^−5^ (@ 1560 nm)
Diamond [36, 39, 46, 47]	2.38	0.22–50	No	2000	0.6 × 10^−5^ (@ 1560 nm)

### 3.2 All-optical NLAF base on femtosecond laser switching of Ge_2_Sb_2_Te_5_

**Figure 3:**
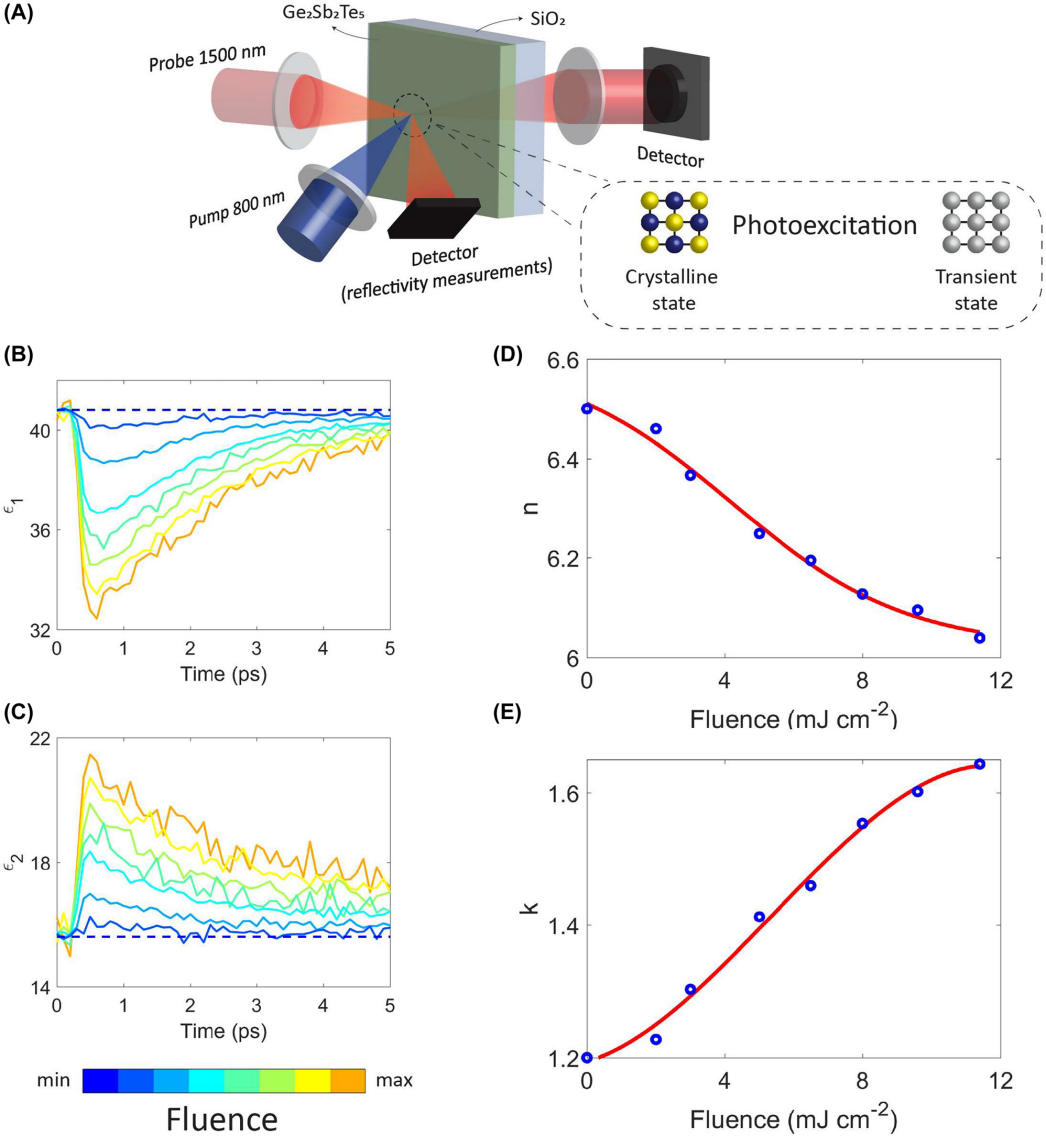
Femtosecond time-resolved pump–probe measurement of crystalline Ge_2_Sb_2_Te_5_. (A) Pump–probe experimental setup. Variation in the (B) real and (C) imaginary part of the dielectric function with time at 1500 nm wavelength when Ge_2_Sb_2_Te_5_ is excited with a 35 fs laser pulse at 800 nm wavelength. Corresponding (D) refractive index, *n*, and (E) extinction coefficient, *k*, of Ge_2_Sb_2_Te_5_ at 1 ps.

A transient change in optical constants is essential for implementing the NLAF. Therefore, we measured the optical properties of crystalline Ge_2_Sb_2_Te_5_ using femtosecond time-resolved pump–probe transmission. Figure 3A shows the pump–probe setup used to switch a crystalline Ge_2_Sb_2_Te_5_ film with a thickness of 27 ± 3 nm. The transient changes to the near-normal reflectivity and transmissivity were simultaneously recorded by a 1500 nm wavelength time-delayed probe with a pump at 800 nm wavelength. The probe was set to 1500 nm wavelength to match the nanophotonic neural network operating wavelength. The dielectric function was then obtained by numerically inverting the Fresnel equations for the multi-layer structure, assuming all changes occurred in the crystalline Ge_2_Sb_2_Te_5_ film. From Figure 3B and C, we observed that the maximum change in the dielectric constants occurred within 1 ps followed by a subsequent relaxation to the original state. The change in optical properties within 1 ps suggests that the material is a good candidate for the NLAF component. Figure 3D and E show the corresponding optical constants at 1 ps for the different laser pump fluence used.

At different wavelengths, the interplay between free carrier absorption and the disruption of metavalent bonds/hyperbonds upon laser excitation have different effects on the crystalline Ge_2_Sb_2_Te_5_ optical response. The free carriers generated from interband transitions lead to free-carrier absorption and thus caused the real part of the dielectric permittivity to decrease and the imaginary part to increase [48]. In contrast, the disruption of metavalent bonds/hyperbond caused both the real and imaginary part of the dielectric permittivity to decrease [15, 49]. Hence the free carrier absorption effect is more pronounced at the 1500 nm wavelength as we see a decrease in the real part of the dielectric function while the imaginary part increases. However, at the 800 nm wavelength the effects of metavalent/hyperbond disruption outweigh free carrier absorption as we see both the real and imaginary part of the dielectric constant decrease [13]. Note, the laser pump in both scenarios were from the same wavelength, at 800 nm.

The transient optical constants of the Ge_2_Sb_2_Te_5_ film, see Figure 3D and E, were incorporated into an AlN micro-ring resonator (MRR). Note, although the material below the Ge_2_Sb_2_Te_5_ film is now AlN, the Ge_2_Sb_2_Te_5_ dielectric constant derived in Figure 3D and E could be used in this analysis. This is because we have isolated the substrate from the Ge_2_Sb_2_Te_5_ film when we used Fresnel equations to derive the Ge_2_Sb_2_Te_5_ dielectric constants. The choice of photonic device can influence the resultant NLAF transfer function. Supplementary Material Section 5 discusses this in further details. In brief, a resonating structure was needed to enhance the nonlinearity of the NLAF component. This is because a straight waveguide structure had a dynamic range of less than 1 dB, as shown in Figure S6 of the Supplementary Material. A NLAF with such a small dynamic range would be susceptible to noise and the signal would need to be amplified. This amplification would increase the overall circuit complexity and power consumption as active amplifiers would be added to each NLAF component.

**Figure 4:**
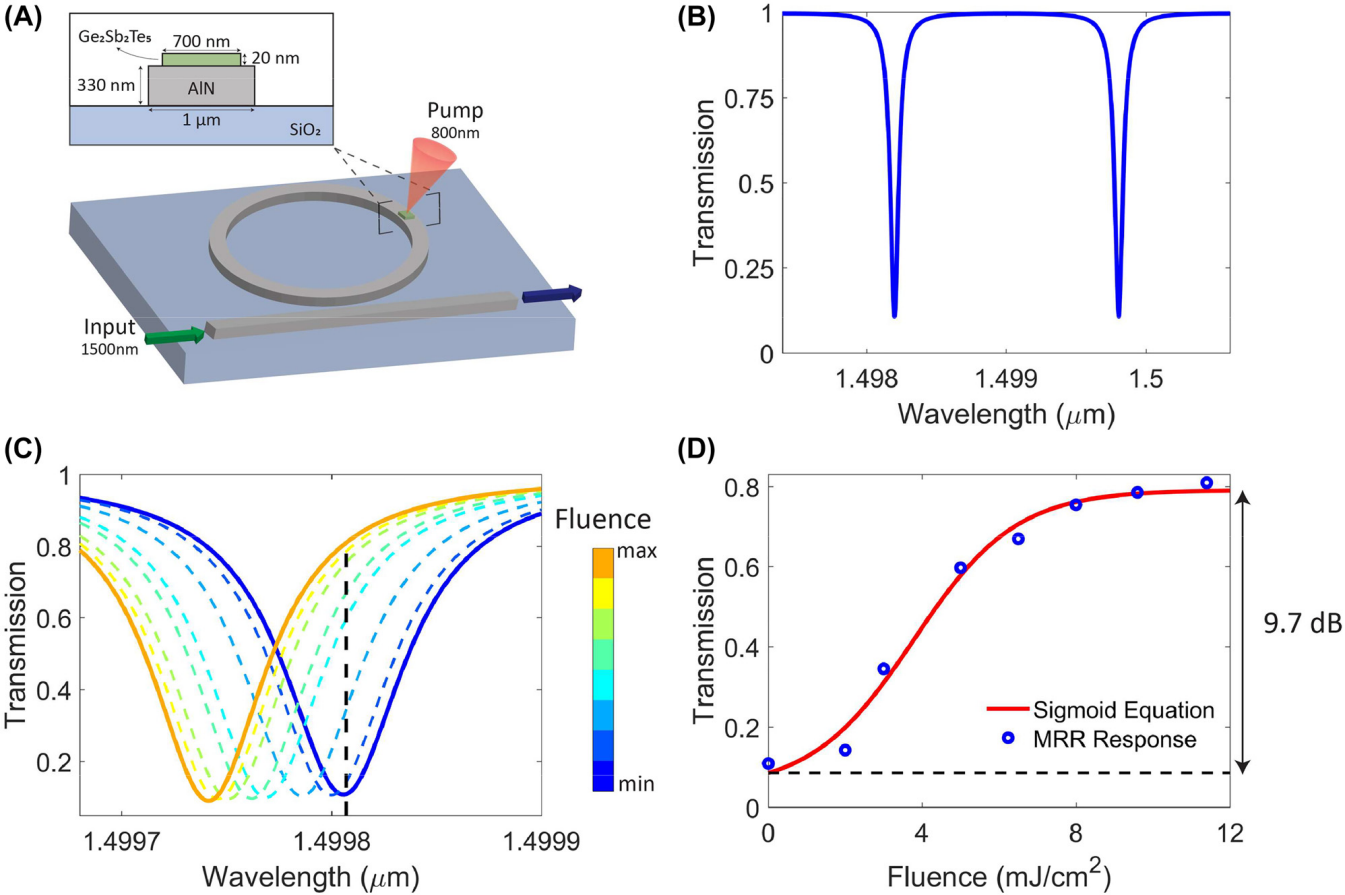
Ge_2_Sb_2_Te_5_-based MRR NLAF. (A) Schematic diagram of MRR with the corresponding dimensions. Spectral response of the Ge_2_Sb_2_Te_5_-based MRR NLAF when it is (B) unperturbed and (C) excited with various femtosecond laser fluence pulses. (D) Resulting sigmoid function used to implement the NLAF. The transmission values of the blue data points intersect with the black dotted line in (C).

By combining the Ge_2_Sb_2_Te_5_ film with a MRR, a sigmoid transmission response with a dynamic range of 9.7 dB was achieved for the NLAF component. The dynamic range was higher than the sigmoid functions reported in reference [10]. Figure 4A shows the Ge_2_Sb_2_Te_5_-based MRR NLAF. The dimensions of the AlN waveguide and Ge_2_Sb_2_Te_5_ film in Figure 4A were optimized to ensure single mode operation at the 1500 nm wavelength. The ring radius was chosen to be 100 µm [26] and the coupling gap between the ring and waveguide was 140 nm to achieve critical coupling. The Ge_2_Sb_2_Te_5_ film length was 100 nm. The spectral response of the photonic device was modeled using Eqs. (3)–(6) to derive the NLAF. Figure 4B and C show the spectrum of the unperturbed device and the device spectral change when excited with various femtosecond laser fluence pulses, respectively. From Figure 4C, the resonance peak of the MRR experienced a blue-shift with increasing laser fluence. This is because the real part of the dielectric function, and thus *n*, of the Ge_2_Sb_2_Te_5_ layer decreased (Figure 3B), which led to a decrease in $n_{\text{eff},\text{GST}}$ found in Eq. (4). This in turn caused the phase shift value, ϕ , in Eq. (4) to decrease thus resulting in a blue-shift in the resonance peak. The blue-shift gave a positive sigmoid function. As the NLAF input is a single wavelength signal, the transfer function of the NLAF will be the change in transmission values for the different laser fluence at a particular wavelength. The sigmoidal NLAF in Figure 4D was obtained by plotting the transmission value of the corresponding laser fluence used at 1499.806 nm wavelength. The black dotted line in Figure 4C intersects the corresponding transmission values of the different laser fluence pulses at the 1499.806 nm wavelength. We chose this wavelength to obtain a positive sigmoid function as the lowest fluence corresponds to the lowest transmission value. Note, the positive sigmoid function is a commonly used NLAF function in machine learning algorithms [50].

The resonance peak amplitude variation of the MRR upon femtosecond laser excitation had similar trends to a PCM-tuned MRR that underwent permanent structural phase transition from the crystalline to amorphous state [51], [52], [53]. We observed a blue shift in the resonance peak as the laser fluence increased. Moreover, the extinction ratio and the *Q*-factor of the device generally increased. Note, this increase is smaller than nonvolatile devices as a permanent structural phase transition caused the Ge_2_Sb_2_Te_5_ material to have a larger change in *n* and *k*. The calculated *Q*-factor and extinction ratio of the MRR for the various laser fluence can be found in Table S4. Although the imaginary part of the Ge_2_Sb_2_Te_5_ material dielectric function, and thus *k*, increased with increasing fluence, the *Q*-factor and extinction ratio still increased. This is because *n* of the Ge_2_Sb_2_Te_5_ layer also decreases, and the mode profile shifts downward in the waveguide. The downward shift was evident when we see a decreasing *n*_eff_ value of the Ge_2_Sb_2_Te_5_-tuned waveguide as the laser fluence increased in Table S2. Moreover, this effect is also illustrated in Figure S3 where we compared the modal profile of the unperturbed MRR and the MRR excited with an 11.4 mJ/cm^2^ laser pulse. As the mode became less confined to the lossy Ge_2_Sb_2_Te_5_ layer, the imaginary part of the effective refractive index decreased. Hence $\alpha_{\text{GST}}$ in Eq. (5) decreases with increasing laser fluence, resulting in a higher *Q*-factor and extinction ratio. As previously mentioned in Section 2, the corresponding $\alpha_{\text{GST}}$ can be found in Table S3. From this result, we observed that the increase in *k* of the Ge_2_Sb_2_Te_5_ material did not compromise the MRR performance. Instead, the *Q*-factor and extinction ratio increased, which was similar, but smaller, to the trends observed in nonvolatile Ge_2_Sb_2_Te_5_-tuned MRR devices.

Implementing the NLAF model on a chip to achieve sub-picosecond optical switching requires cutting-edge PIC technology. Femtosecond on-chip lasers are required for an on-chip NLAF. Current femtosecond switching operations are done in free space and most of them require bulky setups. Nevertheless, developing on-chip femtosecond lasers has started to gain traction [54] and it is likely that this model can be implemented on a chip in the future. In the short term, femtosecond fiber lasers [55] could be used as optical signal inputs, which are small enough to be utilized by data centers.

The computation speed of a feedforward neural network depends on the rate that the NLAF can reset, and this in turn depends on the Ge_2_Sb_2_Te_5_ cooling rate. The heat energy from the Ge_2_Sb_2_Te_5_ layer needs to be dissipated before subsequent pulses can be fired to prevent cumulative heating and subsequent melting. Therefore, we must determine the maximum repetition rate that the crystalline Ge_2_Sb_2_Te_5_ film can be excited.

**Figure 5:**
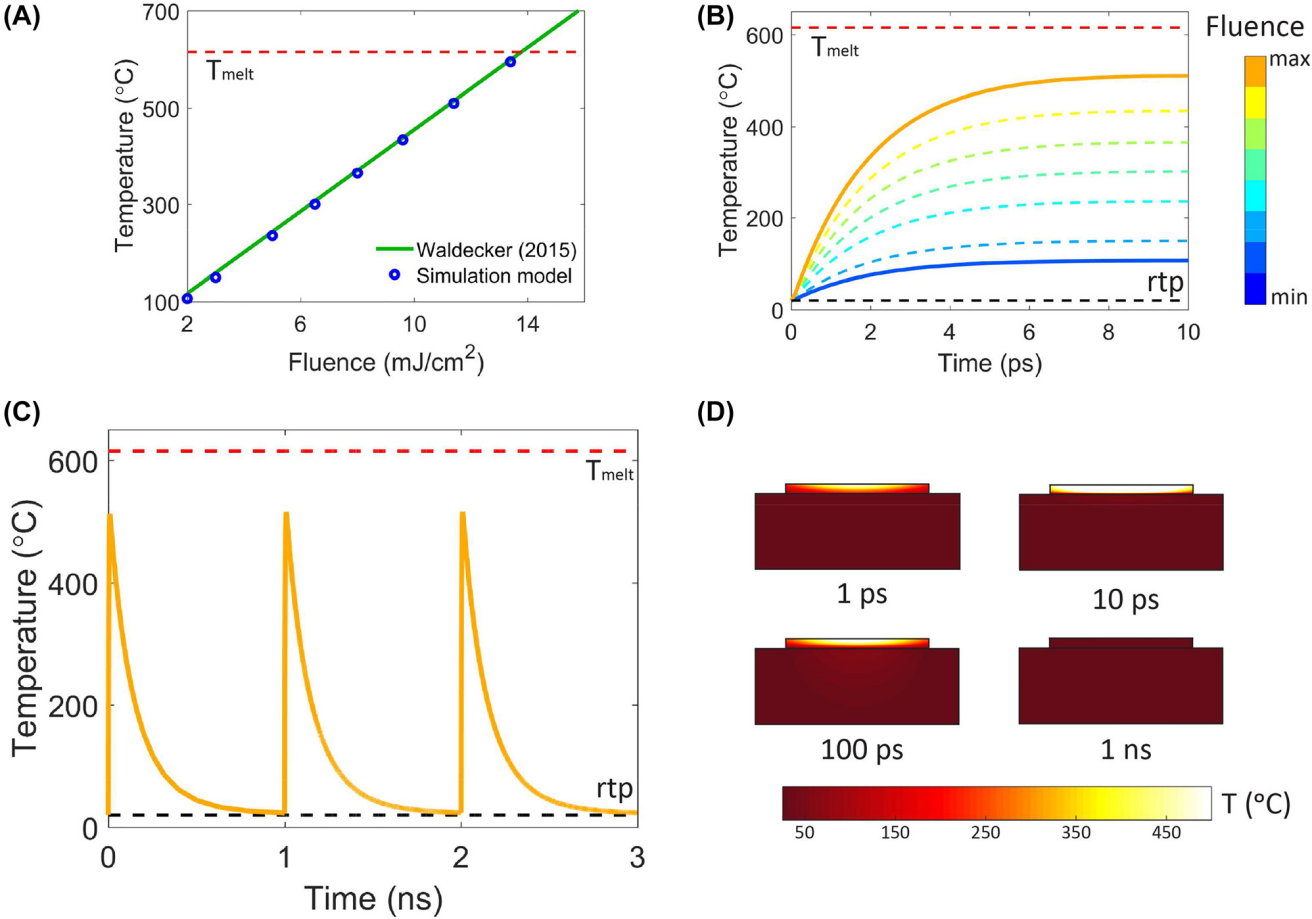
Two-temperature heat simulation of the crystalline Ge_2_Sb_2_Te_5_ material when excited with femtosecond laser pulses. (A) Average temperature of the Ge_2_Sb_2_Te_5_ film after being exposed to the various laser fluence used in Figure 3. (B) Corresponding variation of Ge_2_Sb_2_Te_5_ temperature with time. Both the temperature values and heat dissipation rates match those reported in reference [13]. (C) Average temperature of the Ge_2_Sb_2_Te_5_ film on AlN MRR waveguide when the NLAF is subjected to 1 GHz femtosecond laser pulse train. (D) Heat distribution cross-section of the Ge_2_Sb_2_Te_5_ NLAF MRR at different times after being excited with a 35 fs laser pulse.

A two-temperature model [32, 56] was used to describe the thermal response of Ge_2_Sb_2_Te_5_ during the femtosecond laser switching process. As a femtosecond laser pulse is much shorter than the duration for which the electron and phonons (lattice) temperature equilibrate, there will be a time delay between the laser excitation process and the increase in lattice temperature. This effect is evident in references [13, 56, 57], where the Ge_2_Sb_2_Te_5_ material reaches its equilibrium temperature at the picosecond time scale when excited by a femtosecond laser pulse. The model accounts for this delay by considering the heat transfer from the electrons to lattice during the laser switching process. More information about the two-temperature model analysis can be found in the Supplementary Material Section 6. To validate the simulation model, we first model the femtosecond laser switching experiment of the Ge_2_Sb_2_Te_5_ thin films described in Figure 3A and compared the temperature with measured data by Waldecker et al. [13]. Note, the pump–probe setup and sample were similar in both experiments. The Ge_2_Sb_2_Te_5_ lattice temperature in this work was found to match the temperature values reported by Waldecker et al. Figure 5A compares the simulated temperature and the measured data trendline. The blue data points in Figure 5A were the average temperature across the film surface that was exposed to the laser beam. The temporal change in Ge_2_Sb_2_Te_5_ temperature was also simulated and shown in Figure 5B. When we fitted the time-dependent temperature equation reported by Waldecker et al., we found that the average heating rate across all the laser fluence used was 2.15 ps, with a standard deviation of 0.01 ps. Moreover, the amplitude of the cooling temperature was found to be between 13 and 35% from the increase in temperature. These results were close to the range of values measured by Waldecker et al., where a heating rate of 2.2 ps and cooling temperature range of 20–50% were reported. Thus, this agreement adds a degree of confidence to our heating simulation and concomitant NLAF model.

The two-temperature model was then applied to the NLAF AlN ring resonator structure to determine the maximum repetition rate that the crystalline Ge_2_Sb_2_Te_5_ film can be excited without melting. A train of laser pulses were fired when the material reached room temperature. The laser beam spot was set to 1 um on the waveguide setup. In the simulation, laser pulses were set to the maximum fluence (11.4 mJ/cm^2^) that was needed to program the MRR NLAF. This fluence leads to the slowest operating scenario because the longest cooling time results. Figure 5C shows the average temperature of the Ge_2_Sb_2_Te_5_ film with respect to time when excited with 1 GHz laser pulses at 11.4 mJ/cm^2^. Figure 5D shows the resulting cross-sectional heat distribution in the photonic NLAF MRR at different times. The heat distribution in Figure 5D has a similar switching behavior to that of the thin film sample. At 1 ps, the electrons transfer heat to the lattice, and we see the Ge_2_Sb_2_Te_5_ film temperature increases. The material reaches its equilibrium temperature at 10 ps, after which the heat dissipates into the surrounding. The 100 ps profile exemplifies how heat dissipates from the Ge_2_Sb_2_Te_5_ film. The device reached room temperature at 1 ns. Thus, the AlN waveguide was able to efficiently dissipate the heat, allowing the NLAF component to reset in 1 ns. The NLAF operating speed can be faster when sequential pulses are fired before the Ge_2_Sb_2_Te_5_ film reaches room temperature. However, the structural properties of Ge_2_Sb_2_Te_5_ may change when subjected to long-term cumulative heating and this in turn could alter the behavior of device. Future works should focus on the cyclability of the material and understand how the optical properties of crystalline Ge_2_Sb_2_Te_5_ changes when held above room temperature for long periods of time.

Other ways to improve the operating speed of the NLAF involves optimizing the PCM or introducing redundancy in the circuit. The PCM can be substituted with a chalcogenide that has a higher thermal conductivity. In Figure 2, we show that the heat dissipation is mainly limited by the FCC Ge_2_Sb_2_Te_5_ alloy when the waveguide material is optimized. Hence, chalcogenides with a higher heat conductivity can be considered. For instance, the in-plane heat conductivity of Sb_2_Te_3_ films at room temperature is 5 *W/m⋅K* [58], which is more than twice that of Ge_2_Sb_2_Te_5_ [59]. However, there is a design conflict as a higher thermal conductivity material would also require higher fluence laser pulses to excite the material. Ultimately, the PCM should be optimized to be within the power budget and efficiently dissipate the heat energy generated from the laser pulse. Another solution involves introducing redundancy in the perceptron. Extra NLAF components can be introduced within the perceptron to accelerate the programming process. For instance, introducing three additional NLAF components to one perceptron can enhance programming speed by four times as there will be at least one NLAF component that can be programmed every 250 ps. However, this requires a larger footprint and may not be ideal in large scale networks.

### 3.3 Neural network design and performance

**Figure 6:**
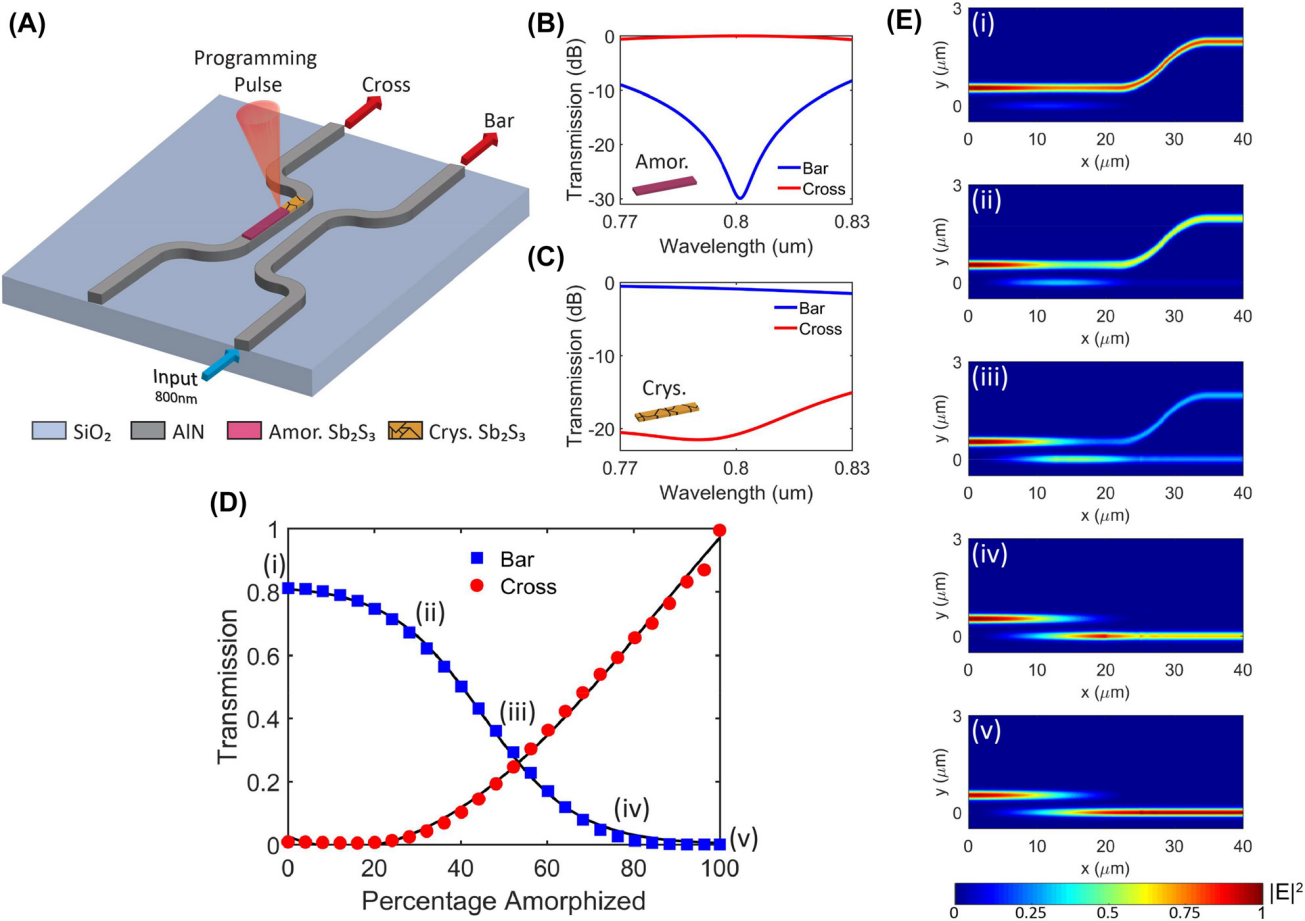
1X2 Sb_2_S_3_-tuned directional coupler for the MZI weights. (A) Schematic diagram of the directional coupler device. Performance of the directional coupler across 770–830 nm wavelength in the (B) amorphous and (C) crystalline state. (D) Transmission values at the different ports for different amorphized Sb_2_S_3_ length at the 800 nm wavelength. (E) Corresponding power field profile of the device for the five states labeled in (D).

An Sb_2_S_3_-tuned directional coupler for the MZI weighting component, shown in Figure 6A, was designed to complement the NLAF. The design method can be found in references [27, 28]. Figure 6B and C show the performance of the AlN directional coupler when Sb_2_S_3_ is in the amorphous and crystalline states, respectively. The directional coupler displayed low insertion losses (<−1.5 dB) and cross talk (−15 dB to −30 dB) for both amorphous and crystalline states in the 770–830 nm wavelength range. Multi-bit switching was introduced by amorphizing 31 sections of the 24.896 µm-long PCM strip. Each section was amorphized at 0.8 µm intervals. Note, the directional coupler has 32 switching states (5 bits) when the purely crystalline state is included. In this work, the focused laser beam, which is used to amorphise the Sb_2_S_3_ sections, is assumed to have a 0.8 µm spot size. However, the strip can also be programmed using a microheater [52, 53, 60, 61]. Microheaters can be programmed to tune the growth of the crystal Sb_2_S_3_ because the crystallization process is growth dominated [28]. Nonplasmonic materials like W make suitable microheater materials as they show minimal insertion losses [62, 63]. Computations done in reference [63] revealed that W microheaters only had an additional insertion loss of 0.1 dB/μm in the TM mode. Note, the position of the W electrodes were also optimized to minimize modal interference. Figure 6D shows the transmission values of the bar and cross ports for different amorphized lengths at the 800 nm wavelength. The corresponding power field profile of the device for the five states labeled as (i)–(v) in Figure 6D, are shown in Figure 6E.

**Figure 7:**
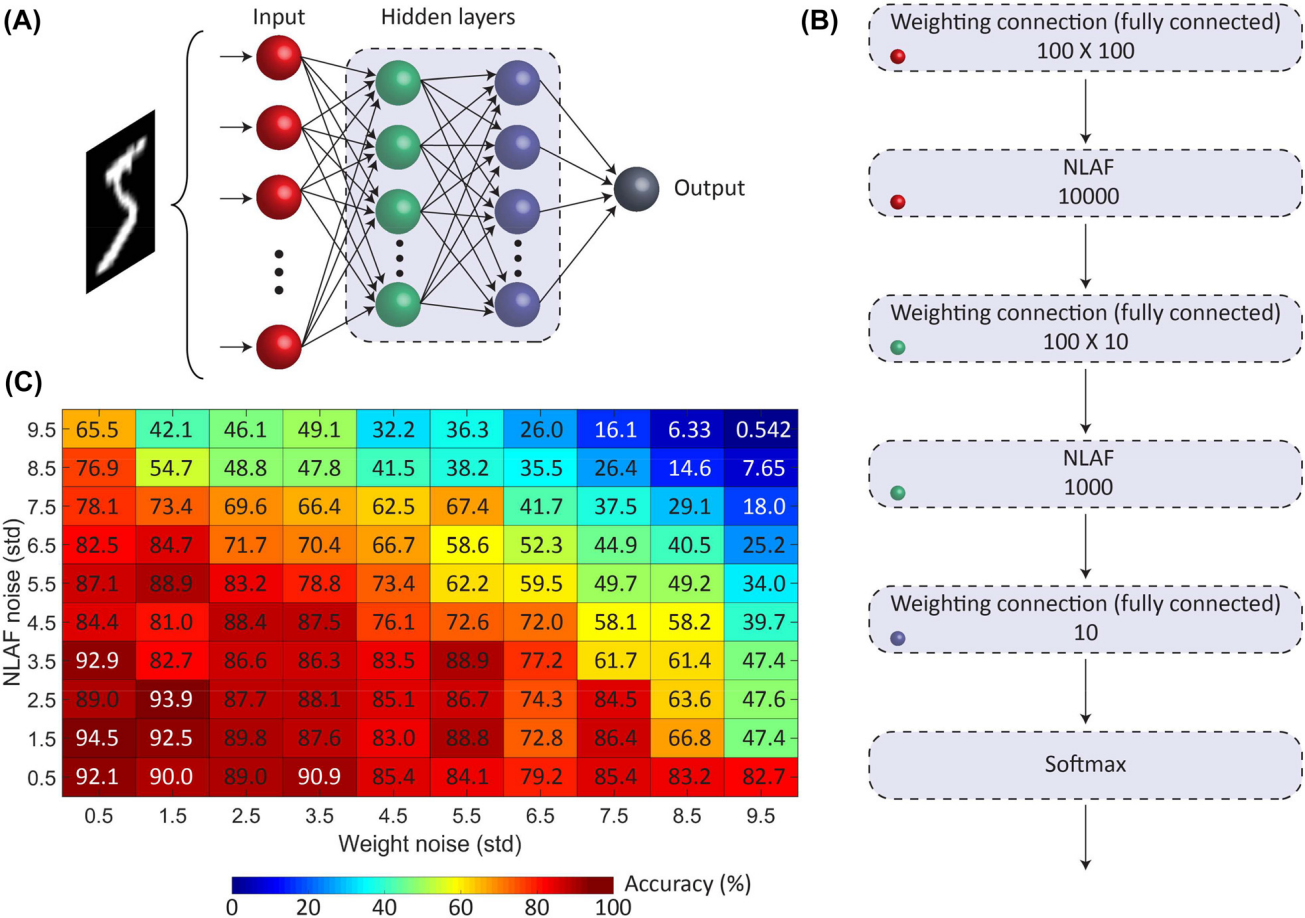
Chalcogenide-tuned neural network used to run MNIST dataset. (A) Schematic of the neural network. (B) Neural network components in each neural layer. The colored circles represent the corresponding neural layers found in (A). (C) Corresponding network accuracy for the different switching errors in the weights and NLAF.

Implementing a multi-layer neural network involves cascading the perceptrons, where each perceptron constitutes the PCM-tuned weights and NLAF. The overall schematic of a three-layer neural network is shown in Figure 7A. Figure 7B shows the neural network components in each neural layer. The colored circles in Figure 7B correspond to the colors used to represent the different neural layers in Figure 7A. Starting from the perceptrons in the first neural layer (red dots), the 800 nm output of the weighting component programs the Ge_2_Sb_2_Te_5_ layer of the NLAF. The 1500 nm output signal from the NLAF then propagates into the second 100 by 100 weighting layer, which is represented by the green dots. To avoid upconversion from 1500 to 800 nm, the weights in the second layer will operate at the 1500 nm wavelength range. The design for the weighting components in the C-band can be found in reference [28]. Upon implementing the weighting computations, the optical signal is then converted back to the electrical domain to implement the rest of the network digitally. Note, the optical signal has to be converted back to the electrical domain to implement the softmax function. Implementing it before the second NLAF layer is ideal as the upconversion of laser signal, which consumes a large amount of power [64], will be avoided. Additional neural layers can be cascaded before the proposed three - layer neural network shown in Figure 7A. The NLAF components in these additional layers will be set to operate at the 800 nm wavelength. This allows the NLAF output signal to propagate directly to the next weighting connection. Moreover, this configuration allows a new set of signals to be generated at each neural layer, specifically at the NLAF input port. The transient optical switching response of Ge_2_Sb_2_Te_5_ measured at 800 nm pump and probe pulses can be found in reference [13]. In this configuration, the penultimate NLAF layer is also set to 1500 nm wavelength and the last NLAF layer is done digitally. Having the penultimate NLAF layer operating at the 1500 nm wavelength is critical as the signal will be integrated with other photonic components involved in the optical–electrical conversion.

The chalcogenide-tuned neural network shown in Figure 7A was modeled to demonstrate its performance. The transfer functions of the components were obtained through curve fitting the data points in Figure 4D and 6D. The NLAF transmission, $T_{\text{NLAF}}$ , as a function of fluence, *F*, is given by Eq. (7). The weights (P^3^A) transmission for the bar, $T_{\text{bar}}$ , and cross, $T_{\text{cross}}$ , ports as a function of the amorphized Sb_2_S_3_ length, *x*, along the coupler are given in Eqs. (8) and (9), respectively. As can be seen, sigmoid and polynomial functions gave the best least-squares fitting results.

(7) $$\begin{array}{c} T_{\text{NLAF}}=\frac{0.7478}{1+2.728\left({\text{e}}^{-0.7522\left(F-2.4486\right)}\right)}+0.04352 \end{array}$$

(8) $$\begin{array}{c} T_{\text{bar}}=\frac{0.8207}{1+0.0139\left({\text{e}}^{0.09293\left(x+0.9209\right)}\right)} \end{array}$$

(9) $$\begin{array}{c} T_{\text{cross}}=-2.845\times 10^{-9}x^{4}-2.92\times 10^{-7}x^{3}+0.0002054x^{2}-0.005326x+0.02582 \end{array}$$

A three-layer fully connected neural network consisting of all-chalcogenide perceptrons was trained on Google TensorFlow with the standard MNIST dataset, which contained 60,000 grayscale images of handwritten digits. The first layer of the network consisted of 100 neurons to receive the image pixels. Similarly, the second layer composed of 100 neurons. Each neuron was connected to all the neurons from the first layer, giving a 100 by 100 connection matrix. The third layer contained just 10 neurons to represent the outputs 0 to 9. The all-optical NLAF (second box in Figure 7B) was connected to the weighting connections found in the first two consecutive neural layers [65].

To test the robustness of this network, we monitor the inference accuracy as a function of switching variability (i.e., noise) from both the P^3^A and the NLAF [66]. Figure 7C shows the corresponding network accuracy for the different noise generated from the P^3^A and NLAF. Unlike digital electronics, where noise is artificially added to the training data to increase network robustness, analogue photonic neural networks have intrinsic noise. The highest inference accuracies were achieved when the standard deviation values were 0.5 and 1.5 for the weights and NLAF, respectively. This corresponds to a Signal to Noise Ratio (SNR) of 6660 for the NLAF and 20,000 for the weights. Hence, it may be possible to deliberately add training noise to fine-tune the network for inference operations on physical noisy input signals. However, we found that when the training noise SNR was less than 2200 for both components (standard deviation values exceeding 4.5), the inference accuracy starts to decrease indefinitely.

The all-chalcogenide all-optical perceptron offers an energy-efficient and fast neural network by avoiding optical to electrical to optical conversions. Only at the penultimate neural layer, the optical signal is converted to the electrical domain to implement the softmax function. The signal remains in the electrical domain after the conversion and further electro-optic conversions are not required. This reduces network latency and energy consumption. Supplementary Material Section 7 and Table S6 compare the network performance with current state of the art electro-optical and all-optical perceptron. The slowest operation time possible of each NLAF is 1 ns, assuming that time is required for the Ge_2_Sb_2_Te_5_ to cool to room temperature before the next pulse is fired, and that all NLAF use the maximum programming fluence of 11.4 mJ/cm^2^. In practice, the NLAF operating speed could be faster as not all NLAFs will need a programming laser fluence of 11.4 mJ/cm^2^. Hence the Ge_2_Sb_2_Te_5_ temperature will not reach its maximum and a shorter time will be required to cool. Moreover, the operating speed can be further increased when subsequent pulses are fired without waiting for the material to cool to room temperature. Since the weights consist of passive devices, the overall network energy is mainly determined by the optical NLAF. This constitutes the femtosecond pulse generation to program the Ge_2_Sb_2_Te_5_ layer and a static input laser signal for the MRR. Note, the NLAF programming pulse is not electrically modulated, and it comes directly from the weighting output. Amplification of the signal may be required but this can be done entirely in the optical domain. For the optical NLAF programming pulse, the maximum energy consumed for the whole network is only 0.90 µJ assuming a total of 10,000 NLAF components, each using the maximum programming fluence of 11.4 mJ/cm^2^ focused to a Gaussian with a beam diameter of 1 µm. The static input signal for the NLAF MRR can be implemented with on-chip lasers, which typically have a high Wall Plug Efficiency (WPE) [67]. For instance, a III-IV based Si on-chip C-band laser can have a WPE of 20% [68, 69]. Assuming that the optical input signal of a NLAF MRR is 1 mW [70], [71], a 100 by 100 weighting connection layer that corresponds to 10,000 NLAF components would require an electrical input of 50 W to power the lasers. This is substantially lower than von Neumann technologies like a GPU, which has a power consumption of up to 300 W [72]. The Sb_2_S_3_-tuned MZI is nonvolatile and can preserve the optical weights. Thus, no additional power is required when performing the weighting function under the feedforward propagation mode.

## 4 Conclusion

To conclude, by combining AlN waveguides with two different effects in chalcogenides, we show that a passive, efficient, and accurate all-optical perceptron is feasible. For the first time, we demonstrate that the disruption of delocalized *p*-orbital metavelent bonds with a femtosecond laser can be used to implement a photonic NLAF device with sub-picosecond dielectric permittivity switching response. Moreover, the low–loss Sb_2_S_3_ material used to set the network interconnection MZI weights gave low insertion loss and cross talk. The proposed all-optical perceptron model can also be extended to the terahertz frequency in the future, as a recent study demonstrated volatile and nonvolatile switching of Ge_2_Sb_2_Te_5_ [73]. In the feedforward mode, the heat dissipation of the NLAF material is the bottleneck of the network. To improve the NLAF efficiency, future works should study the cyclability of Ge_2_Sb_2_Te_5_ material switching in the femtosecond time range and how the thermal properties of the material changes when it accumulates heat energy. The speed of the NLAF can also be improved by considering other PCMs, like Sb_2_Te_3_, which has a higher thermal conductivity, or by optimizing the architecture of the network to accommodate multiple NLAF components. Ultimately, this depends on the design requirements, particularly the network power budget and chip footprint.

## Supplementary Material

Supplementary Material
