# Supplementary material for: Programmable chalcogenide-based all-optical deep neural networks
Source: Nanophotonics. 2022 May 25;11(17):4073–88. doi: 10.1515/nanoph-2022-0099 (PMC11501810; doi:10.1515/nanoph-2022-0099)
Supplement: Supplementary file 1 — Supplementary Material [file j_nanoph-2022-0099_suppl.docx]

Ting Yu Teo^2†*^, Xiaoxuan Ma^1†^, Ernest Pastor^3^, Hao Wang^2^, Jonathan K. George^1^, Joel K. W. Yang^2^, Simon Wall^3,4^, Mario Miscuglio^1^, Robert E. Simpson^2^*, Volker J. Sorger^1^*

^1^ Deptartment of Electrical and Computer Engineering, George Washington University, Washington DC, DC, USA

^2^ Singapore University of Technology and Design, 8 Somapah Road, 487372 Singapore, Singapore

^3^ICFO - Institut de Ciencies Fotoniques, The Barcelona Institute of Science and Technology, Av. Carl Friedrich Gauss 3, 08860 Castelldefels (Barcelona), Spain

^4^Department of Physics and Astronomy, Aarhus University, Ny Munkegade 120, 8000 Aarhus C, Denmark

^†^These authors contributed equally to this work

Corresponding authors: t[ingyu](mailto:simon.wall@phys.au.dk)_teo@mymail.sutd.edu.sg, [robert_simpson@sutd.edu.sg](mailto:robert_simpson@sutd.edu.sg), [sorger@gwu.edu](mailto:sorger@gwu.edu)

Programmable Chalcogenide- based All - Optical Deep Neural Networks: Supporting Material

1. Reflectivity measurements of the Ge_2_Sb_2_Te_5_ film to determine phase transition temperature


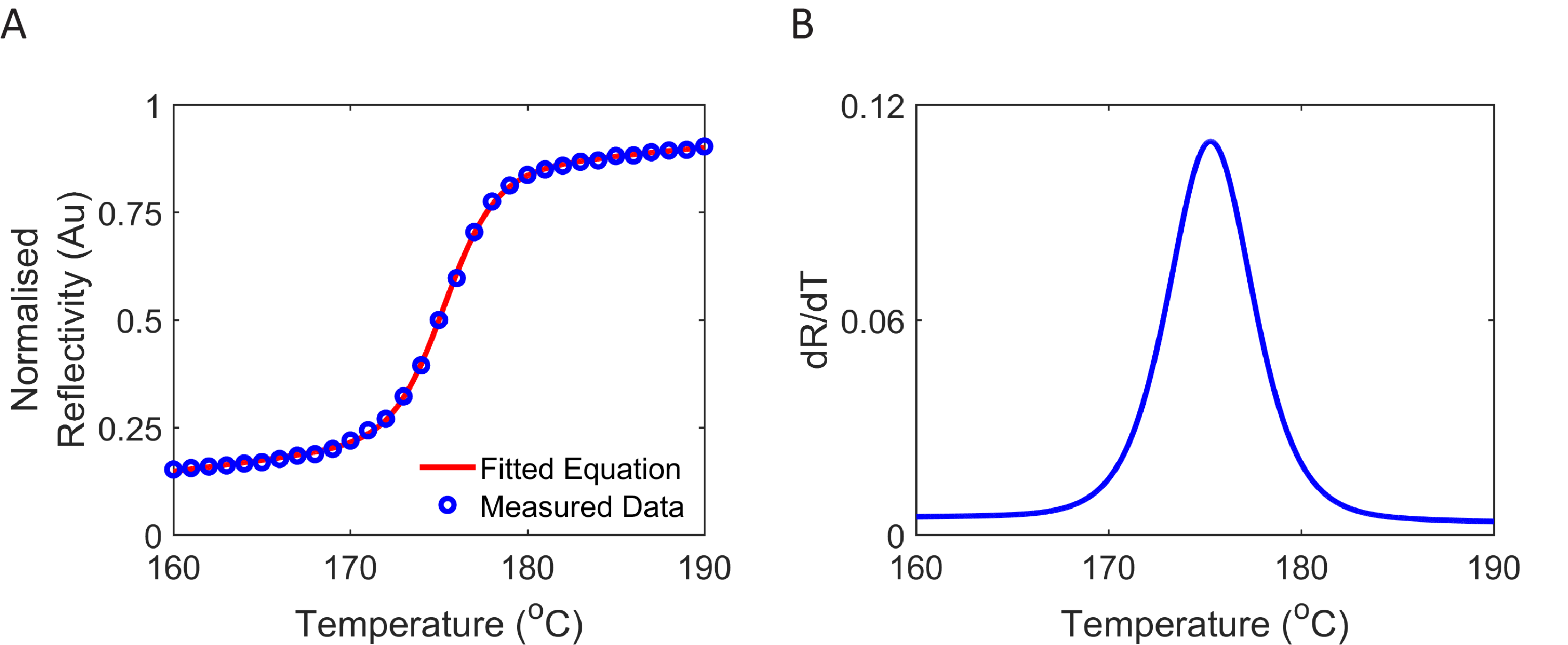


Figure S1: Reflectivity measurements of Ge_2_Sb_2_Te_5_ thin films to determine the phase transition temperature. (A) Change in reflectivity with respect to the change in temperature. (B) Differential of (A). A sigmoid function was fitted into (A) to simplify the differential operation.

The reflectivity data points in Figure S1A were fitted with a sigmoid equation. Note, we refrain from interpreting any physical meaning from the equation’s coefficients and simply use it to find the peak rate of crystallisation. The sigmoid equation was differentiated to determine the phase transition rate as a function of temperature. Figure S1B shows the differentiated curve, with the peak rate of crystallization occurring at 176 $℃$. This corresponds to the phase transition temperature. Upon determining the phase transition to be 176 $℃$, we crystallized the Ge_2_Sb_2_Te_5_ thin film at 183 $℃$ with a heating ramp rate of 5 $℃$ /s and hold time of 30 minutes. The resultant film was used for the pump-probe femtosecond switching experiments.

1. Ge_2_Sb_2_Te_5_ - tuned MRR NLAF modelling parameters

To calculate the spectrum of the MRR, $n_{eff}$ and $\alpha$ in Eqs. (4) and (5) of the main text were required. Tables S1 to S3 show the $n_{eff, AlN}$ , $n_{eff, GST}$ and $\alpha_{GST}$ of the waveguides. Note that as a region of the MRR is deposited with a layer of Ge_2_Sb_2_Te_5_, the effective refractive index ($n_{eff, GST}$) of the Ge_2_Sb_2_Te_5_- tuned waveguide is required. The $n_{eff}$ values vary with wavelength, and we found that a small change can affect the positions of the resonance peak. To account for this variability, we fit a linear equation across the wavelength of 1480 nm to 1520 nm:

$$\begin{aligned} n_{eff}=a*\lambda+b\#\left( S1 \right) \end{aligned}$$

With $a$ and $b$ being constants. Note, $\lambda$ is in units of $\mu$m.

Table S1 and S2 show the coefficient values of the linear equation in Eq. (S1). $\alpha_{GST}$ values in Table S3 are the Ge_2_Sb_2_Te_5_ - tuned waveguide absorption at 1500 nm. The values were obtained by solving Maxwell’s equation using Lumerical mode solution. The full wave simulation accounted for scattering, which is critical at the Ge_2_Sb_2_Te_5_ - tuned region. The schematic of the simulation model is found in Figure S2 and the mode profile of the Ge_2_Sb_2_Te_5_ on AlN waveguide for laser fluence 0 mJ/cm^2^ and 11.4 mJ/cm^2^ can be found in Figure S3. We observed that with higher laser fluence, the mode is less confined to the Ge_2_Sb_2_Te_5_ layer as its refractive index decreases.

Table S1: Coefficients for $n_{eff, AlN}$

|  | No PCM |
| --- | --- |
| a | -0.3955 |
| b | 2.228 |

Table S2: Coefficients for $n_{eff, GST}$

| Fluence | 0 | 2 | 3 | 5 | 6.5 | 8 | 9.6 | 11.4 |
| --- | --- | --- | --- | --- | --- | --- | --- | --- |
| a | -0.5092 | -0.4941 | -0.458 | -0.4138 | -0.3965 | -0.3681 | -0.3547 | -0.3445 |
| b | 2.62 | 2.588 | 2.512 | 2.418 | 2.381 | 2.321 | 2.292 | 2.267 |

Table S3: $\alpha_{GST}$ values

| Fluence | 0 | 2 | 3 | 5 | 6.5 | 8 | 9.6 | 11.4 |
| --- | --- | --- | --- | --- | --- | --- | --- | --- |
| $\alpha$ at 1500nm wavelength (dB/cm) | 73938.1 | 73519.3 | 72805.6 | 71818.7 | 71022.7 | 70904.1 | 70765.8 | 69294.8 |


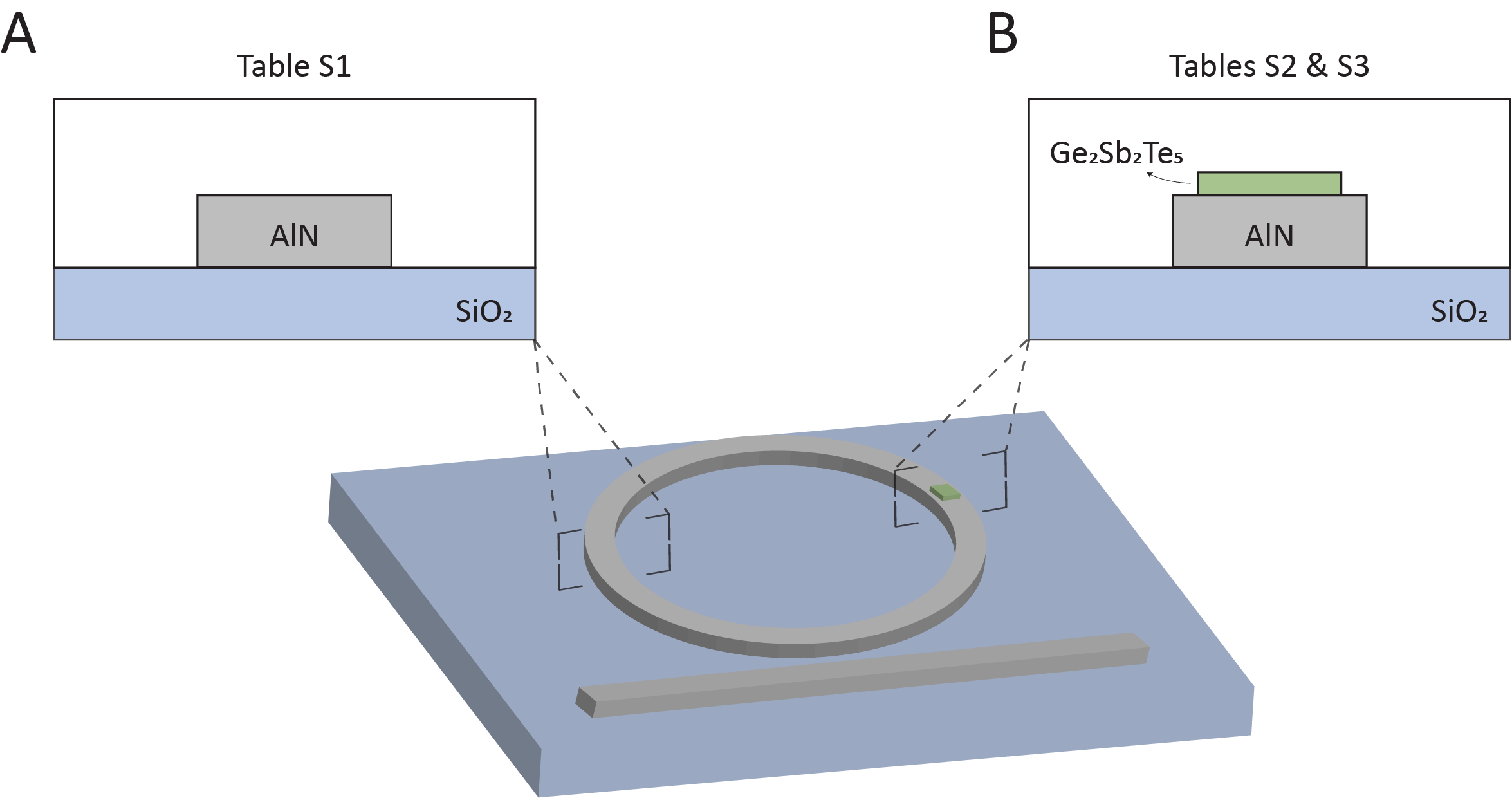


Figure S2: Schematic of the waveguide structure used in simulation to obtain (A) $n_{eff, AlN}$ , (B) $n_{eff, GST}$ and $\alpha_{GST}$ of the waveguides


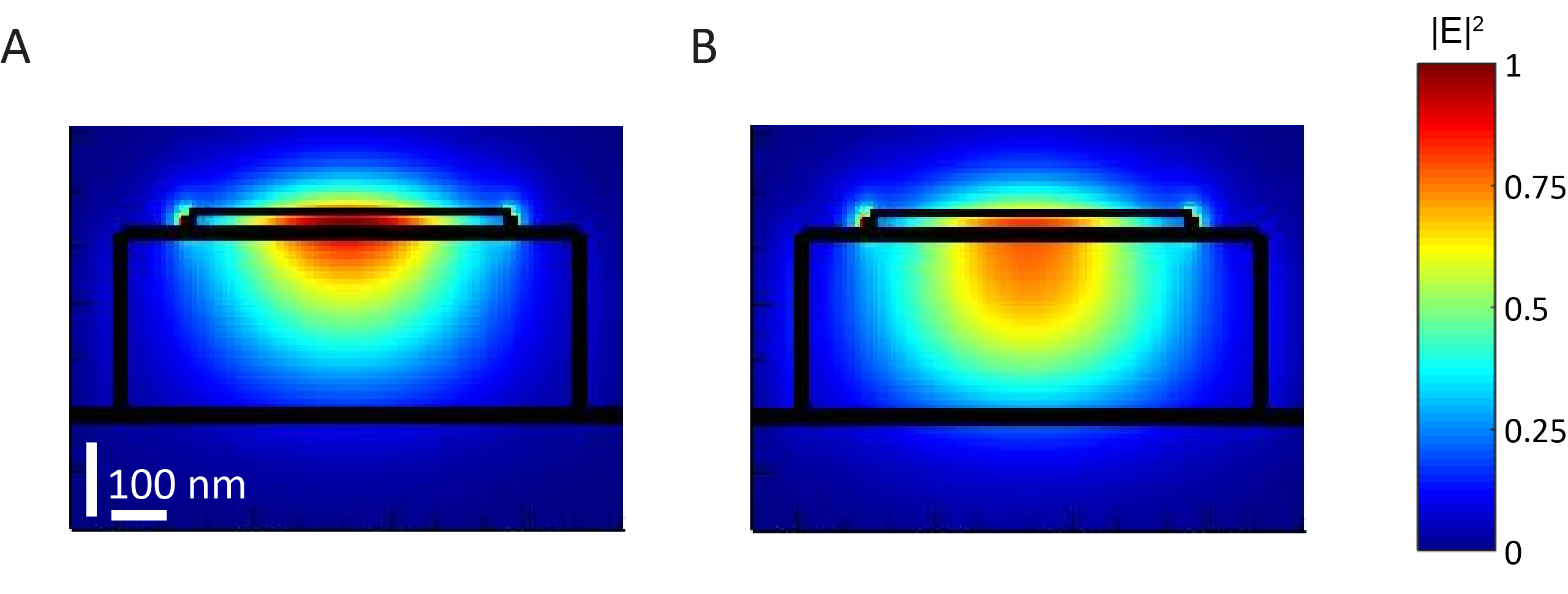


Figure S3: Mode profile of the Ge_2_Sb_2_Te_5_ on AlN waveguide for laser fluence (A) 0 mJ/cm^2^ and (B) 11.4 mJ/cm^2^

Upon obtaining the spectral response of the various laser fluences, we then show the corresponding Q factor and extinction ratio of the MRR in Table S4. The Q values were derived from the graph in Figure 4C by using Eq. (S2) [1]:

$$\begin{aligned} Q= \frac{\lambda_{res}}{FWHM} \#\left( S2 \right) \end{aligned}$$

Table S4: Q factor and extinction ratio of MRR response with varying laser fluence

| Fluence | 0 | 2 | 3 | 5 | 6.5 | 8 | 9.6 | 11.4 |
| --- | --- | --- | --- | --- | --- | --- | --- | --- |
| Q | 21500 | 21600 | 21700 | 21900 | 22100 | 22100 | 22100 | 22300 |
| Extinction Ratio (dB) | 9.70 | 9.76 | 9.87 | 10.02 | 10.15 | 10.18 | 10.20 | 10.45 |

1. Sb_2_S_3_ – tuned directional coupler model

The directional coupler was designed to vary the phase matching conditions of the two coupled waveguides base on the PCM structural phase. Figure S4 shows the even and odd mode profile of the two waveguides during phase matching and non-phase matching conditions. The phase matching condition in Figure S4A corresponds to the amorphous phase for Sb_2_S_3_ while the non-phase matching condition in Figure S4B is in the crystalline state. Following the design methods in [2, 3], we optimized the directional coupler dimensions as shown in Figure S5.


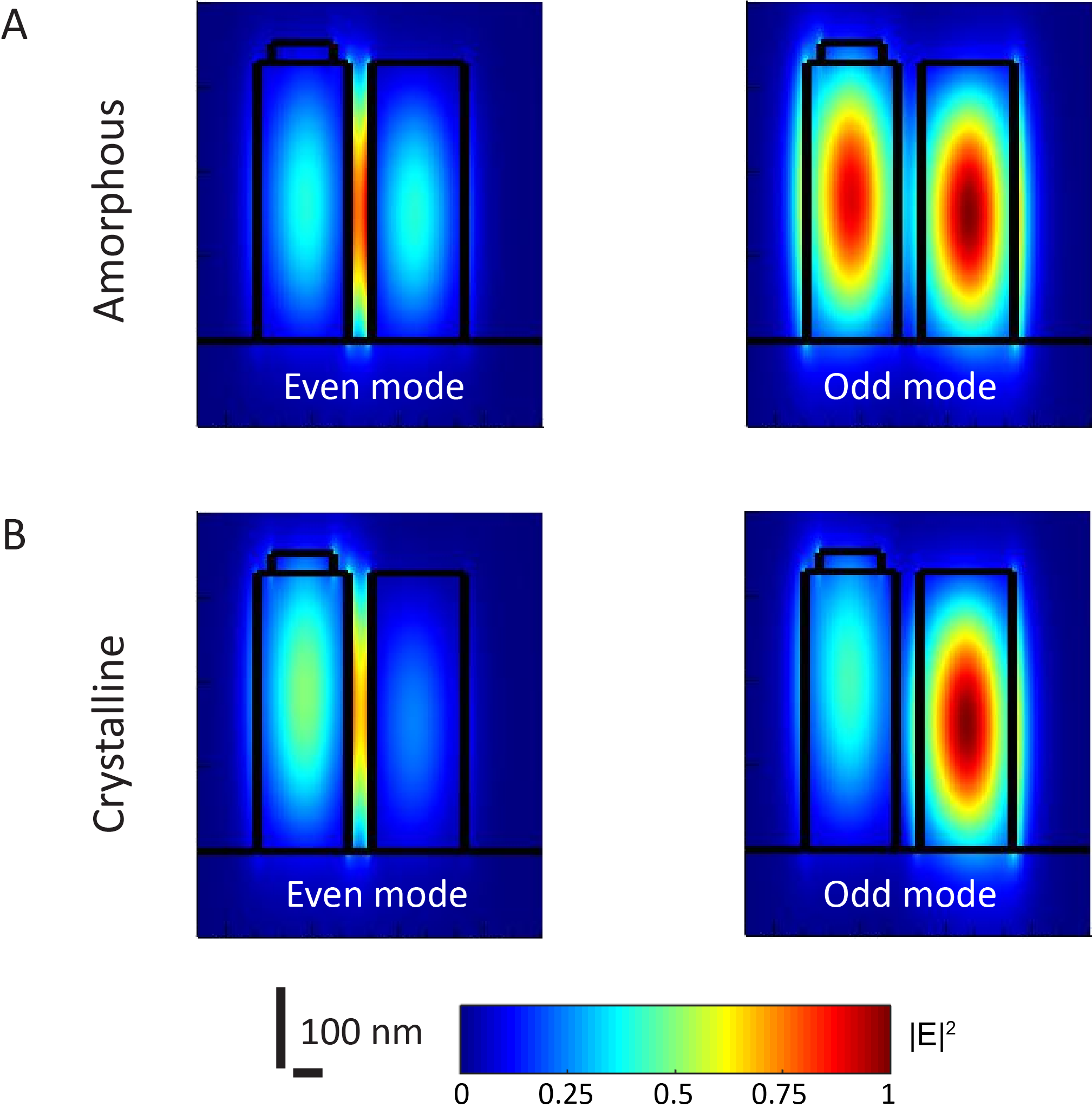


Figure S4: Mode profile of the directional coupler during (A) phase and (B) non-phase matching conditions in the amorphous and crystalline state respectively.


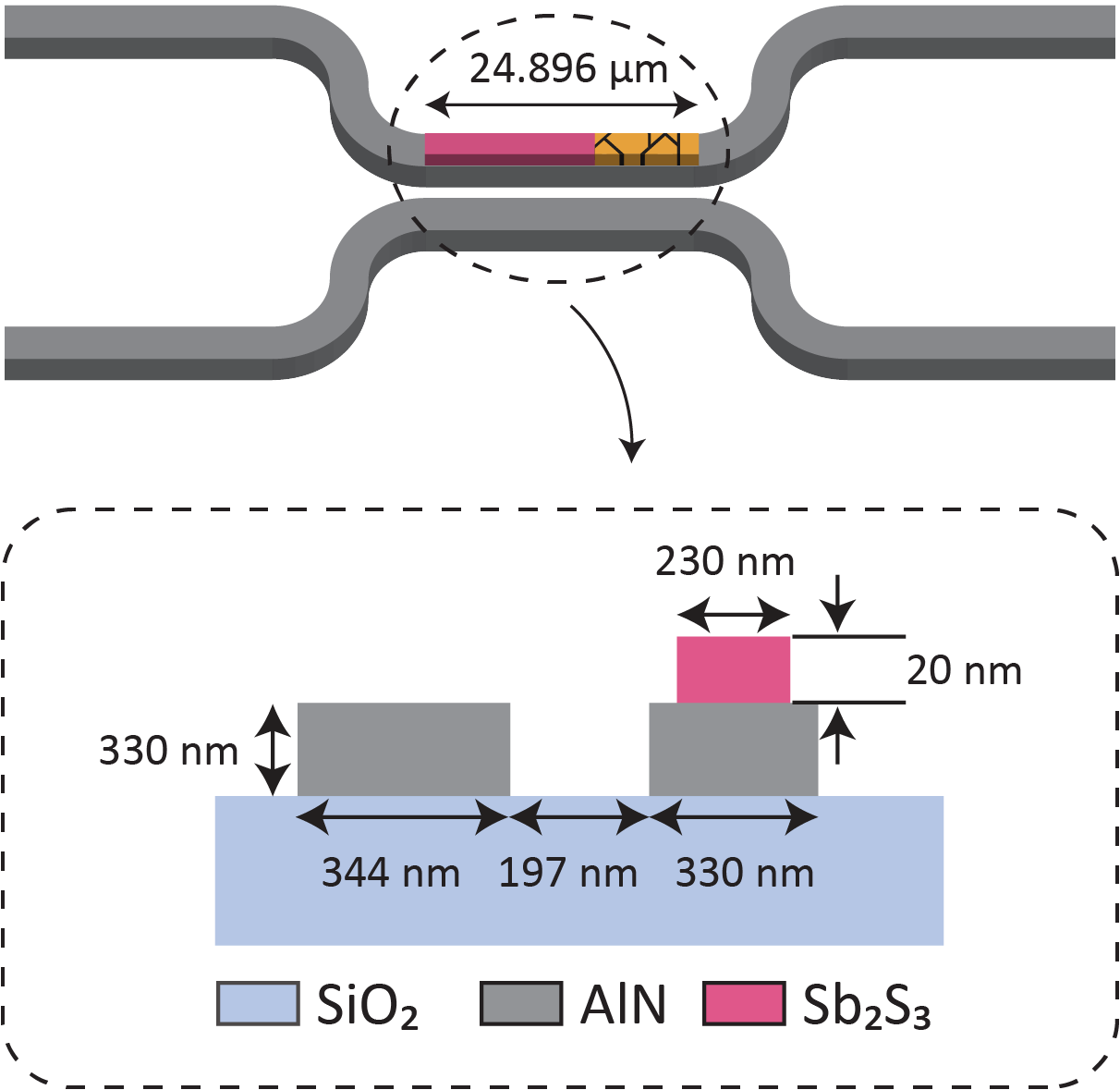


Figure S5: Optimized directional coupler dimensions.

1. Waveguide material comparison model parameters

Finite element method from COMSOL was used to determine the heat dissipation rates of the four waveguide materials. Table S5 shows the waveguide material thermal parameters used in the simulation model. The thermal conductivity values from Table 1 of the main text was also required for this simulation model.

Table S5: Waveguide material thermal parameters used in simulation model

| Material | Si | SiN | AlN | Diamond |
| --- | --- | --- | --- | --- |
| Heat capacity (J/(kg*K)) | 700 [4] | 700 [5] | 3300 [6] | 516 [4] |
| Density (kg/m^3^) | 2329 [7] | 3100 [8] | 1002.78 [9] | 3515 [10] |

1. Influence of photonic device on NLAF


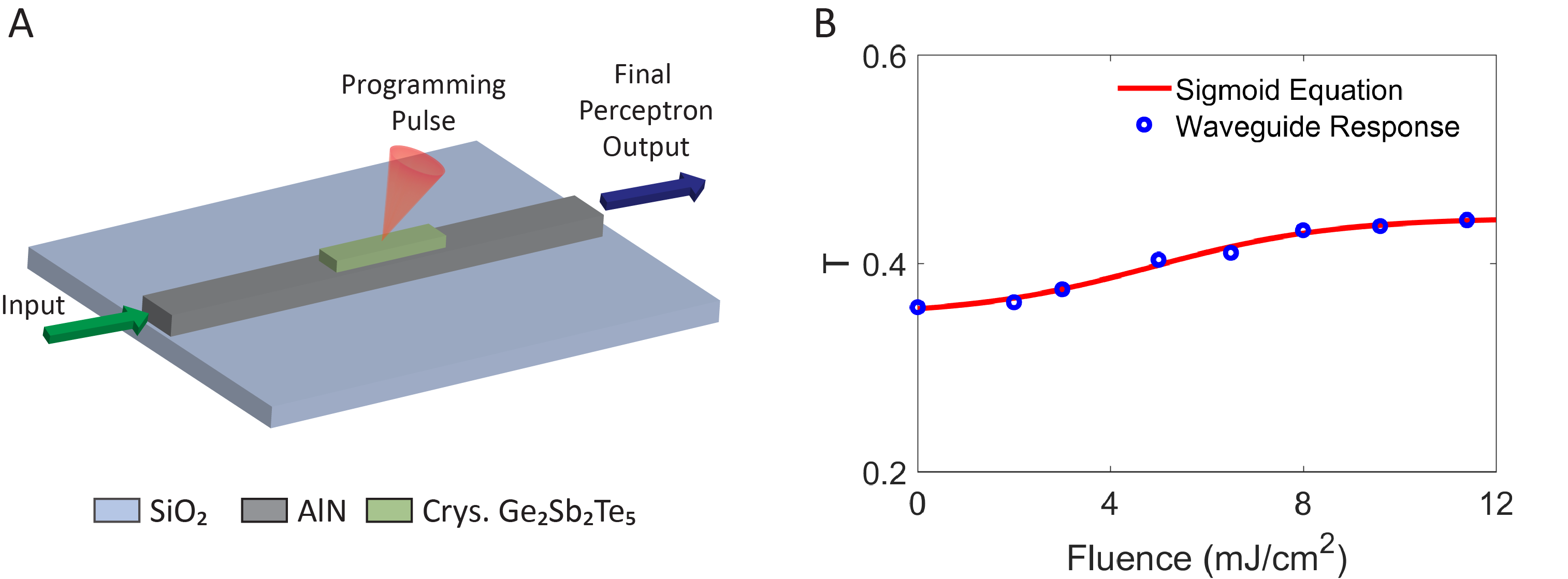


Figure S6: Ge_2_Sb_2_Te_5_ – tuned straight waveguide NLAF. (A) Schematic and (B) transfer function of a Ge_2_Sb_2_Te_5_ – tuned straight waveguide.

The choice of photonic device can influence the resultant NLAF transfer function. We compare the NLAF response of a Ge_2_Sb_2_Te_5_– tuned straight waveguide and the Ge_2_Sb_2_Te_5_– tuned MRR implemented in the main text. The Ge_2_Sb_2_Te_5_– tuned straight waveguide NLAF is shown in Figure S6A and its corresponding transfer function is shown in Figure S6B. The NLAF dynamic range is less than 1 dB. From the two transfer functions, Figure 4D and S6B, the dynamic range of the MRR is higher than the straight waveguide. This is due to the nature of the photonic device. The resonating structure results in resonance peaks and introduced a large transmission disparity between the minimum and maximum laser fluence at a single wavelength.


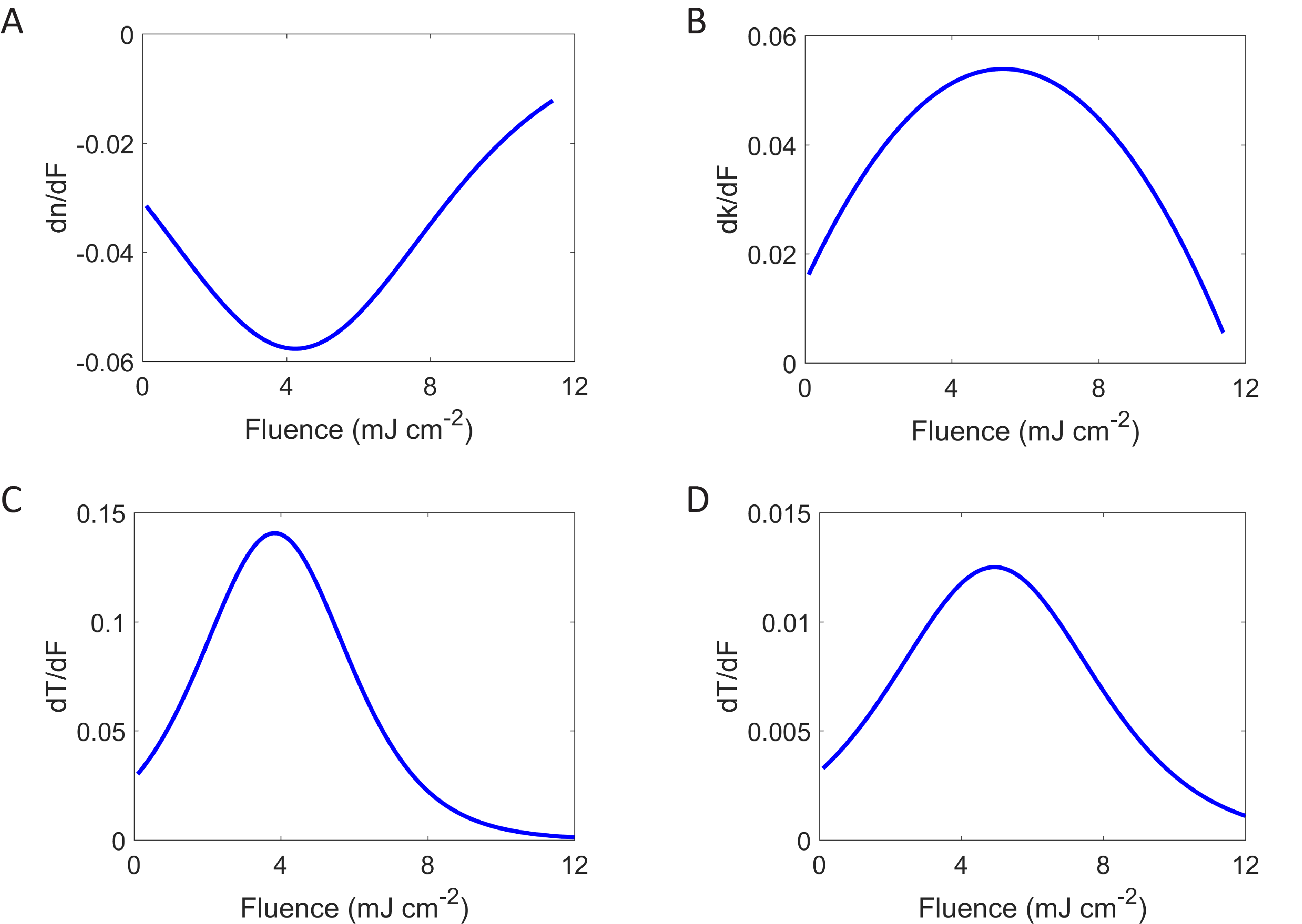


Figure S7: Comparison of change in Ge_2_Sb_2_Te_5_ n and k with change in T (transmission) for the different photonic devices. Differential curves of (A) n of Ge_2_Sb_2_Te_5_ (Figure 3D), (B) k of Ge_2_Sb_2_Te_5_ (Figure 3E), (C) Ge_2_Sb_2_Te_5_ – tuned MRR (Figure 4D), and (D) Ge_2_Sb_2_Te_5_ – tuned straight waveguide (Figure S6B).

Besides the dynamic range, the choice of photonic device also influenced the shape of the sigmoid curve. We differentiated the two sigmoidal curves in Figure 4D and S6B to compare the change in transmission with the change in n and k values for the different laser fluence used (Figure 3D and E). Figure S7A to D show the differential of: (i) $n$ and $k$ of Ge_2_Sb_2_Te_5_ in Figure 3D and E, (ii) Sigmoid function of the MRR (Figure 4D) and (iii) Sigmoid function of the straight waveguide (Figure S6B). From the Ge_2_Sb_2_Te_5_ n and k differential curves in Figure S7A and B, we see that the maximum change is between the laser fluence of 4 to 6 mJ/cm^2^. This has influenced the maximum change of transmission for both the Ge_2_Sb_2_Te_5_ – tuned photonic devices to also be around this region. The straight waveguide maxima (Figure S7D) is closer to the $k$maxima (Figure S7B) because the transmission is mainly governed by the Beer Lambert’s law [11]. Conversely, the MRR maxima (Figure S7C) is closer to the $n$ minima (Figure S7A). This is because the change in transmission of the MRR is caused by the phase shift of the MRR spectral response. This phase shift is dependent on the change in $n_{eff}$ of the Ge_2_Sb_2_Te_5_ region in the ring (Eq. (4) main text), which in turn is dependent on the change in $n$ of the Ge_2_Sb_2_Te_5_ material. Next, we observed that the MRR transmission saturates after 9.6 mJ/cm^2^. This is due to the MRR spectral response. With increasing fluence, the resonance peak experienced a blue shift, and the corresponding transmission value quickly moves away from the minima of the resonance peak. This caused the NLAF output to saturate at a faster rate than the straight waveguide output and we see that the change in transmission tend towards zero for high fluences. From these results, we demonstrate that the equations governing the photonic devices mainly influenced the resulting NLAF transfer function. Moreover, these equations are dependent on the optical constants of the Ge_2_Sb_2_Te_5_ material.

1. Two temperature model analysis of the fs switching operation

The two-temperature model used to determine the temperature of the Ge_2_Sb_2_Te_5_ film during the fs switching operation can be mathematically represented as [12]:

$$\begin{aligned} \rho C_{e}\frac{\partial T_{e}}{\partial t}=\nabla\left( k_{e}\nabla T_{e} \right)-\gamma\left( T_{e}-T_{l} \right)+Q\#\left( S3 \right) \end{aligned}$$

$$\begin{aligned} {\rho C}_{l}\frac{\partial T_{l}}{\partial t}=\nabla\left( k_{l}\nabla T_{l} \right)+\gamma\left( T_{e}-T_{l} \right)\#\left( S4 \right) \end{aligned}$$

where $\rho$ is the density of the material, $C$ is the heat capacity, $k$ is the heat conductivity, $T$ is the temperature, $\gamma$ is the electron-phonon coupling coefficient, $Q$ is the heat energy absorbed by the material and $t$ is the time. The subscripts $e$ and $l$ represents the electrons and lattice respectively.

The energy density absorbed by the thin film is expressed as:

$$\begin{aligned} \Theta=F\alpha\left( 1-R \right)e^{\frac{-2r^{2}}{{\omega_{r}}^{2}}}e^{\left( -\alpha z \right)}\#\left( S5 \right) \end{aligned}$$

where $F$ is the laser fluence, $R$ is the reflectance of the thin film, $\alpha$ is the absorption coefficient, $\omega_{r}$ is the pulse waist radius (1/e^2^). $r$ and $z$ are the cylindrical coordinates of the Ge_2_Sb_2_Te_5_ thin film, with $r$ being the radial distance from the center of the beam spot exposed on the film and $z$ along the thickness of the film.

Considering the laser pulse duration, the power density absorbed by the thin film is expressed as:

$$\begin{aligned} \Lambda=\frac{\Theta}{\sqrt{\pi}\tau}e^{-\frac{\left( t-2\tau\right)^{2}}{\tau^{2}}}\#\left( S6 \right) \end{aligned}$$

$\tau$ is pulse duration and for a Gaussian pulse, $\tau$ is expressed as:

$$\begin{aligned} \tau=\frac{t_{pulse-width}}{2\sqrt{ln2}}\#\left( S7 \right) \end{aligned}$$

The two-temperature model was implemented in COMSOL using the finite element method. In our simulation model the Ge_2_Sb_2_Te_5_ material parameters are as follows: $\rho=6270 kg/m^{3}$ [13], $C_{l}=250 J/(kg*K)$ [14]. $k$ is expressed as: $k= 0.0006T+1.1144, 27℃<T<615℃.$The trend is based on the measurements previously reported in reference [15]. The change in $k$ with temperature was accounted for in the model as it was known to affect the temperature value of Ge_2_Sb_2_Te_5_ [15]. We assumed that $k_{e}=k_{l}$ whilst $C_{e}$ was estimated to be ten times lower than $C_{l}$ [16]. We estimate the electron – phonon coupling coefficient to be $7\times{10}^{16}$. For the heat energy absorbed by the material, $F$ were the fluence values used in the laser switching experiment, ranging from $2 mJ/cm^{2}$ to $11.4 mJ/cm^{2}$. $\alpha=\frac{4\pi*img(N)}{\lambda}$ , where $img(N)$ is the imaginary part of the effective refractive index of the material which corresponds to the extinction coefficient. From ellipsometry measurements,$img(N)=3.776$ at $800 nm$. $\lambda$ was set to the pump wavelength, $800 nm$. $R$ at $800 nm$ wavelength was found to be $0.63$ upon converting the dielectric constant values of the Ge_2_Sb_2_Te_5_ film to the corresponding $R$ values by solving Fresnell’s equation using the transfer matrix method. $t_{pulse-width}$ was 35 fs.

The thermal properties of the AlN waveguide and SiO_2_ substrate were also required in the model. The thermal properties of the AlN waveguide can be found in Table S5 and Table 1 of the main text. The SiO_2_ thermal properties are as follows: $k$ [17], $\rho$ [18, 19] and $C$ [20, 21].

1. Comparison with state-of-the-art optical neural networks

We compare the all-optical perceptron model with current state-of-the-art computing technology. The results are summarized in Table S6. It shows that (1) the optical to electrical to optical (O-E-O) conversion can increase network latency and power consumption and (2) active devices are power hungry and can limit network scalability. Electrical NLAF networks require O-E-O conversion at each neural layer. The conversions are inefficient as they increase latency and introduce additional signal losses. The first conversion is for the weighting output (optical) to pass through the NLAF (electrical) and the second conversion is for the resulting NLAF signal (electrical) to pass through the next weighting components (optical). Moreover, it is essential to use passive devices for the neural network components. We observe that active devices like the thermo-optic MZI weights and optical amplifiers in the NLAF can increase power consumption exponentially when large scale networks are implemented.

Table S6: Comparison of the all-Chalcogenide perceptron model with current state of the art computing technology.

| Hardware  /Model | Weighting Component | | NLAF Component | | Power Limitation  (Feedforward network) | Delay Limitation  (Feedforward network) | Network Accuracy |
| --- | --- | --- | --- | --- | --- | --- | --- |
|  | Dom-ain | Resolution | Dom-ain | Type |  |  |  |
| GPU | E | 32 bits | E | All | Communication with Memory + Operations | Digital NLAF (> 0.1 ns) + Communication with Memory (ms) | > 98 %  (MNIST) |
| MZI-based Photonic Neural Network [22] | O | 16 bits | E | ReLu | Optical input signal + 10 mW for each MZI to hold optical state + O - E - O conversion | PD** + Digital NLAF (> 0.1 ns) + Communication /w Computer Memory (ms) | 90* %  (Vowel sound recognition) |
| PCM Photonic Tensor Core [23] | O | 5 bits | E | ReLu | Optical input signal + O - E - O conversion | PD** + Digital NLAF (> 0.1 ns) + Communication /w Computer Memory (ms) | 95.3 %  (MNIST) |
| All optical perceptron (WDM Weights + SOA- MZI NLAF) [24] | O | 2 bits | O | Sigm-oid | Optical input signal + Weights (four modulators) + $\geq$ 10 mW for each NLAF | PD** + Optical weights (1.6 ns) + Optical NLAF (1.6 ns) | Unknown, not tested |
| This work | O | 5 bits | O | Sigm-oid | Optical input signal + NLAF (maximum 0.90 $\mu$J for entire network + 50 W for 10000 NLAF laser input signal at each neural layer) | PD** + Optical NLAF ($\leq$ 1 ns) + digital NLAF only for the last layer ($<$0.1 ns) | 94.5 %  (MNIST) |

* This value was projected from one MZI device and not the overall network. The final accuracy value was lower when the whole network was demonstrated. However, for a fair comparison with our perceptron model, we have used the training accuracy value from one MZI device.

**Photodetector is used to convert from the optical to electrical signal at the penultimate or last neural layer to implement the Softmax function electrically.

1. References

1. W. Bogaerts, P. De Heyn, T. Van Vaerenbergh et al., "Silicon microring resonators," *Laser & Photonics Reviews* **6**(1), 47-73 (2012).

2. T. Y. Teo, M. Krbal, J. Mistrik, J. Prikryl, L. Lu, and R. E. Simpson, "Comparison and analysis of phase change materials-based reconfigurable silicon photonic directional couplers," *Opt. Mater. Express* **12**(2), 606-621 (2022).

3. P. Xu, J. Zheng, J. K. Doylend, and A. Majumdar, "Low-Loss and Broadband Nonvolatile Phase-Change Directional Coupler Switches," *ACS Photonics* **6**(2), 553-557 (2019).

4. S. A. Hedayat Mofidi, A. Khounsary, and F. Mashayek, "Thermo-mechanical properties of silicon, germanium, diamond, beryllium and silicon carbide for high heat load x-ray optics applications," *Proceedings of SPIE - The International Society for Optical Engineering* **8502**((2012).

5. M. Gastreich, J. D. Gale, and C. M. Marian, "Charged-particle potential for boron nitrides, silicon nitrides, and borosilazane ceramics: Derivation of parameters and probing of capabilities," *Physical Review B* **68**(9), 094110 (2003).

6. J. A. W. Shackelford, *CRC Materials Science and Engineering Handbook, Third Edition*, CRC Press (2000).

7. P. Zhou, R. Chen, N. Wang, H. San, and X. Chen, "Reliability Design and Electro-Thermal-Optical Simulation of Bridge-Style Infrared Thermal Emitters," *Micromachines* **7**(166 (2016).

8. W. M. Haynes, *CRC Handbook of Chemistry and Physics, 92nd Edition*, CRC Press, Hoboken (2011).

9. C. Xiong, W. H. P. Pernice, X. Sun, C. Schuck, K. Y. Fong, and H. X. Tang, "Aluminum nitride as a new material for chip-scale optomechanics and nonlinear optics," *New Journal of Physics* **14**(9), 095014 (2012).

10. J. R. D. K. Asmussen, *Diamond films handbook*, Marcel Dekker, New York (2002).

11. D. F. Swinehart, "The beer-lambert law," *Journal of chemical education* **39**(7), 333 (1962).

12. H. Liu, W. Dong, H. Wang et al., "Rewritable color nanoprints in antimony trisulfide films," *Science Advances* **6**(51), eabb7171 (2020).

13. W. K. Njoroge, H.-W. Wöltgens, and M. Wuttig, "Density changes upon crystallization of Ge2Sb2.04Te4.74 films," *Journal of Vacuum Science & Technology A* **20**(1), 230-233 (2002).

14. L. Waldecker, T. A. Miller, M. Rude et al., "Time-domain separation of optical properties from structural transitions in resonantly bonded materials," *Nat Mater* **14**(10), 991-995 (2015).

15. M. Kuwahara, O. Suzuki, Y. Yamakawa et al., "Temperature Dependence of the Thermal Properties of Optical Memory Materials," *Japanese Journal of Applied Physics* **46**(6B), 3909-3911 (2007).

16. X. Sun, M. Ehrhardt, A. Lotnyk et al., "Crystallization of Ge2Sb2Te5 thin films by nano- and femtosecond single laser pulse irradiation," *Scientific Reports* **6**(28246 (2016).

17. D. W. Lee, and W. D. Kingery, "Radiation Energy Transfer and Thermal Conductivity of Ceramic Oxides," *Journal of the American Ceramic Society* **43**(11), 594-607 (1960).

18. P. S. T. C. T. E. Gaal, "Thermal conductivity 24/Thermal expansion 12 : Joint conferences : [proceedings of the Twenty-Fourth International Thermal Conductivity Conference] : [proceedings of the Twelfth International Thermal Expansion Symposium"]."

19. T. A. Hahn, and R. K. Kirby, "Thermal Expansion of Fused Silica from 80 to 1000 K ‐ Standard Reference Material 739," *AIP Conference Proceedings* **3**(1), 13-24 (1972).

20. R. B. Scott, *Cryogenic engineering*, D. Van Nostrand, Princeton (1959).

21. L. H. Van Vlack, *Physical ceramics for engineers*, Addison-Wesley Pub. Co., Reading, Mass. (1964).

22. Y. Shen, N. C. Harris, S. Skirlo et al., "Deep learning with coherent nanophotonic circuits," *Nature Photonics* **11**(7), 441-446 (2017).

23. J. Feldmann, N. Youngblood, M. Karpov et al., "Parallel convolutional processing using an integrated photonic tensor core," *Nature* **589**(7840), 52-58 (2021).

24. G. Mourgias-Alexandris, A. Tsakyridis, N. Passalis, A. Tefas, K. Vyrsokinos, and N. Pleros, "An all-optical neuron with sigmoid activation function," *Opt. Express* **27**(7), 9620-9630 (2019).
